# Spatially Resolved Strain Mapping In Flexible Oxide Membranes

**DOI:** 10.1002/smll.74801

**Published:** 2026-07-23

**Authors:** Pol Salles, Marti Ramis, Eric Brand, Peter Kúš, Julie Ruellou, Andrea Sartori, José Manuel Caicedo Roque, Dae‐Sung Park, Nini Pryds, Mariona Coll, Jakub Drnec

**Affiliations:** ^1^ European Synchrotron Radiation Facility (ESRF) Grenoble France; ^2^ Institut de Ciència de Materials de Barcelona (ICMAB‐CSIC) Cerdanyola del Vallès Spain; ^3^ Department of Mining, Industrial and ICT Engineering Universitat Politècnica de Catalunya (UPC) Manresa Spain; ^4^ Department of Energy Storage and Conversion Technical University of Denmark Kongens Lyngby Denmark; ^5^ Department of Surface and Plasma Science Faculty of Mathematics and Physics Charles University Prague Czech Republic; ^6^ ALBA Synchrotron Barcelona Spain; ^7^ Catalan Institute of Nanoscience and Nanotechnology (ICN2) CSIC and BIST Bellaterra Spain

**Keywords:** La_0.7_Sr_0.3_MnO_3_, oxide membranes, strain, synchrotron, XRD

## Abstract

Strain engineering in single‐crystalline oxide membranes offers a versatile platform to tailor functional properties beyond thin films or bulk materials. However, accurately determining strain transfer and distribution within these membranes remains challenging, limiting direct correlations between structural distortion and functional response and hindering the rational design of flexible devices. Here, high‐resolution synchrotron x‐ray diffraction is used for in situ monitoring of strain in (001)‐oriented ${\mathrm{La}}_{0.7}{\mathrm{Sr}}_{0.3}{\mathrm{MnO}}_{3}$ membranes on flexible polymer substrates under stretching and bending. This approach enables quantitative determination of in‐plane strain as both macroscopic averages and spatially resolved maps. Macroscopic analysis shows that strain transfer is most efficient in thinner films and leads to distinct strain symmetries under stretching and bending. Spatially resolved measurements reveal local strain heterogeneity that increases with applied stress beyond macroscopic averages. Correlating strain distribution with estimated Curie temperature shifts illustrates how such heterogeneity could translate into spatially non‐uniform functional behavior. Additionally, the setup's sensitivity to crystallographic orientation enables analysis of stacked and twisted architectures, where interlayer strain transfer is effective yet attenuated with distance from the substrate. Altogether, this framework establishes a robust approach for probing oxide membranes, opening pathways toward strain‐engineered flexible devices, multilayer heterostructures, and operando investigations.

## Graphical abstract

High‐resolution synchrotron x‐ray diffraction reveals how strain transfers and distributes within crystalline oriented oxide membranes (10–100 nm thick) on flexible substrates that are subjected to stretching and bending. The study uncovers local strain heterogeneities that deviate from macroscopic averages, providing a crucial framework when correlating structural distortion with functional response and paving the way for the rational design of flexible electronics.

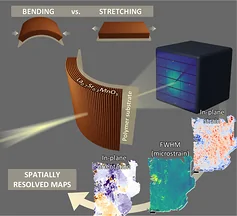

## 1 Introduction

Strain engineering in complex oxide materials has emerged as a powerful strategy to control and enhance their rich spectrum of functional properties [1]. In these correlated systems, mechanical deformation is intimately coupled to the charge, spin, and orbital degrees of freedom, as even small variations in lattice symmetry can profoundly alter the underlying electronic structure and phase behavior [2, 3]. This makes strain a uniquely versatile tuning parameter, capable of stabilizing metastable states and driving emergent functionalities not accessible in bulk materials. Traditionally, epitaxial strain arising from the lattice mismatch between thin films and their underlying crystalline substrates has been the primary route to modulate these properties [4, 5, 6], facilitating significant discoveries such as strain‐induced ferroelectricity [7] or enhanced magnetotransport [8]. However, the range of strain achievable in epitaxial films is inherently constrained by the finite lattice parameter mismatch with the underlying substrates, thereby limiting access to a wider range of strain tunability in these films [9, 10, 11].

By releasing the epitaxial thin films from their rigid and brittle substrates, freestanding oxide membranes offer an exceptional platform for strain engineering, enabling large, continuously tunable, and versatile strain states [12, 13]. For instance, uniaxial tensile strains exceeding 8% have been demonstrated in ${\mathrm{La}}_{0.7}{\mathrm{Ca}}_{0.3}{\mathrm{MnO}}_{3}$ (LCMO) on polymeric substrates [14], and up to 10% in ${\mathrm{BaTiO}}_{3}$ freestanding membranes [15], highlighting the possibility of highly tunable strains in freestanding forms. Moreover, the oxide membranes can be deformed into distinct strain states including uniaxial, biaxial, linear, curved, and even strain‐gradient configurations. This capability overcomes the symmetry constraints inherent to epitaxial growth and opens new avenues for manipulating the physical properties of complex oxides [12, 14, 16]. For example, anisotropic bandgap tuning has been demonstrated in ${\mathrm{BiFeO}}_{3}$, resulting in both an increase and a decrease of the bandgap when uniaxial strain is applied along the pseudocubic [100] and [110] crystallographic directions, respectively [17]. This versatility in strain engineering is determined by the approach used to apply the mechanical stress.

The most common strategies to control strain include: (i) multilayer epitaxy of freestanding films, where lattice mismatch across layers induces strain [18, 19]; (ii) mechanical bending, which allows reversible tuning of tensile and compressive strain [20]; and (iii) mechanical stretching, which allows continuous and directional control of uniaxial or biaxial tensile strain [14, 21]. Additionally, the resulting strain also depends on whether the membrane is freestanding or supported by a flexible substrate. For instance, in freestanding membranes, bending can induce strong strain gradients across the film thickness ‐ similarly to the strain gradients obtained in wrinkled membranes ‐ [15, 16], while when bending flat membranes on supported configurations, the much larger thickness of the substrate results in nearly uniform strain with negligible gradient along the membrane thickness [12].

In practice, strain transfer in oxide membranes is a complex phenomenon. The fabrication of large‐area, defect‐free membranes remains challenging, as cracks, wrinkles, and other imperfections are often introduced during film release or transfer [22, 23]. To mitigate these effects, several strategies have been proposed, including the use of sacrificial layers with lattice parameters closely matched to those of the functional oxide films [24, 25], deliberate epitaxial strain engineering in both the sacrificial layers and the oxides during heterostructure growth [26], and engineered transfer stamps designed to accommodate strain relaxation during the lift‐off process (e.g., elastically graded polymers) [27, 28]. Even when the oxide membranes are successfully transferred onto external target platforms (e.g., silicon and flexible substrates), strain transfer between the membrane and targeted substrate is far from straightforward: it depends on their relative Young's moduli, adhesion strength, and the elastic limits of both materials [22]. Moreover, non‐uniform stress fields in the flexible substrate or at the membrane edges can lead to spatially varying strain, whose characteristics depend on the applied stress mode [14, 29, 30]. Beyond single‐layer oxide membranes, strain can play a critical role in interfacial coupling and twistronics in artificially stacked oxide membranes with controlled twisting angles [31, 32, 33], yet the mechanisms governing strain transfer and accommodation within such systems remain largely unexplored. Therefore, understanding and quantifying strain transfer and its spatial distribution is essential for reliably correlating structural deformation with functional responses in oxide membranes.

These factors highlight the need to probe strain with high precision and spatial resolution. Most current approaches rely on area‐averaged measurements or indirect mechanical estimations [11, 34], which overlook local variations and the intrinsic heterogeneity of real samples. Electron‐based techniques such as STEM ‐ including nanobeam electron diffraction (NBED) and dark‐field imaging modes ‐ can achieve nanometer spatial resolution and have been used to map strain in 2D materials and single‐crystalline oxide membranes [35, 36], but require electron‐transparent samples, are limited to small fields of view, and cannot be readily combined with macroscopic mechanical loading. X‐ray diffraction (XRD) is the standard technique for crystallographic strain determination, offering non‐destructive access with high accuracy. However, conventional laboratory XRD provides only macroscopic averages determined by the beam footprint, thus missing the local strain variations that arise at the microscale. Furthermore, the weak diffraction signal from thin membranes has so far limited true in situ or operando strain measurements, restricting most studies to ex situ characterization at discrete deformation states. Synchrotron‐based high‐resolution XRD offers a unique opportunity to overcome these limitations, combining high brilliance and sensitivity with spatial resolution and operando capability [37, 38]. Scanning x‐ray diffraction microscopy has emerged as a powerful route for mapping strain in thin films, using focused beams with spatial resolutions ranging from a few micrometers down to the nanometer scale [19, 39], and dedicated in situ loading stages have extended this capability to mechanically deformed samples [40, 41]. Yet, in situ spatially resolved XRD strain mapping across millimeter‐sized single‐crystalline oxide membranes under controlled mechanical loading has not been reported.

Here, we systematically present a case study exploring strain transfer in (001)‐oriented ${\mathrm{La}}_{0.7}{\mathrm{Sr}}_{0.3}{\mathrm{MnO}}_{3}$ (LSMO) membranes, a strongly correlated perovskite oxide whose magnetic, electric and catalytic properties are highly sensitive to structural deformations [2, 8, 34, 42]. The LSMO membranes, supported on flexible polymer substrates, were mounted on a custom‐built strain stage, where mechanical stress was applied either through stretching or bending of the substrate. Using synchrotron‐based high‐resolution XRD in transmission geometry with a tunable beam size, we probed both macroscopic averages (∼9000 $\mu m^{2}$) and spatially resolved (∼90 $\mu m^{2}$ pixel size) in‐plane lattice strains across the membrane surface, allowing us to visualize how strain distributes within millimeter‐scale membranes. By systematically varying stress mode (bending vs. stretching) and membrane thickness (10–100 nm), we identify the key parameters that govern efficient strain transfer and its symmetry. Furthermore, we illustrate the relevance of probing the strain distribution to understand the resulting functional response by estimating the local variations in Curie temperature arising from the measured strain heterogeneity. Finally, we demonstrate the potential of this approach for probing in‐plane orientation with high resolution and resolving strain in stacked and twisted membranes. Altogether, our findings reveal that strain transfer in oxide membranes is far more intricate than typically assumed, underscoring the need to account for spatial strain distribution when designing strain‐engineered and flexible oxide devices and heterostructures.

## 2 Results and Discussion

**FIGURE 1:**
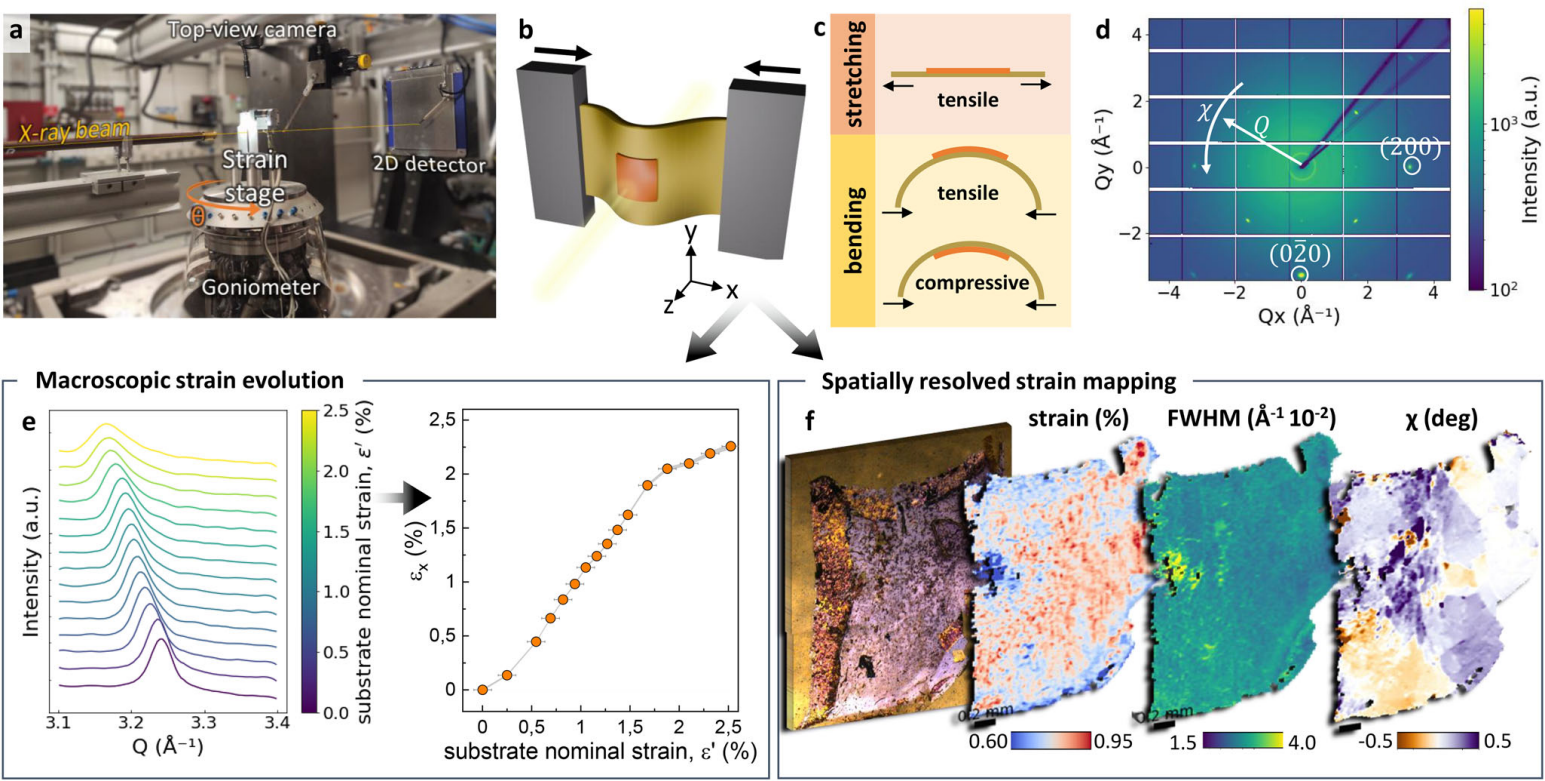
Setup configuration for strain engineering measurements. (a) synchrotron HRXRD setup, including the integrated strain stage for in situ mechanical loading, a goniometer stage for sample alignment, a top‐view camera and a 2D detector. (b) Illustration of the strain stage geometry used to apply strain with in situ measurements in transmission. The oxide membrane is represented in orange and the polymer substrate in yellow. (c) Mechanical stress modes used in the experiment. (d) Raw 2D detector image for a 10 nm LSMO membrane on PI. (e) Macroscopic strain evolution: Peak intensity of the (200) reflection as a function of Q (Å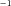) under different substrate nominal strain ($\varepsilon^{\prime }$) (left); corresponding measured strain ($\varepsilon_{x}$) plotted as a function of $\varepsilon^{\prime }$ (right). (f) Spatially resolved strain mapping: optical microscope image of the membrane (left) and maps of $\varepsilon_{x}$ strain, FWHM $Q_{\left(200\right)}$, and membrane orientation χ (right). Scale bar of 200 μm.

To investigate strain transfer in oriented oxide membranes, we developed a synchrotron‐based high‐resolution XRD (HRXRD) setup equipped with an in situ mechanical strain stage (Figure 1a). The (001)‐oriented LSMO membranes were fabricated by selective water etching of epitaxial heterostructures, as described elsewhere [22, 24], transferred to polyimide (PI) flexible substrates, and then mounted with the *a* lattice parameter (*a*‐LP) direction aligned with the direction of the mechanical stress (Figure 1b). Mechanical stress was applied either by stretching or bending (tensile/compressive)(Figure 1c), and the resulting substrate nominal strain ($\varepsilon^{\prime }$) was determined from optical measurements of the substrate elongation or bending radius, respectively (Figure S1). Diffraction patterns were acquired in transmission geometry, with the x‐ray beam passing through membrane and substrate [37]. Thanks to the extremely brilliant source of the ESRF‐EBS and a high‐energy beam, even membranes as thin as 10 nm yielded well‐defined Bragg peaks from the in‐plane reflections (Figure 1d). The resulting diffraction data were then treated so individual Bragg peaks (200) and (020) were isolated and fitted with Gaussian‐shaped peaks (Figure S2). This analysis yields three key parameters: (i) the peak position along the scattering vector Q, which provides the in‐plane lattice parameters (*a*‐LP and *b*‐LP) and the corresponding strain ($\varepsilon_{x}$ and $\varepsilon_{y}$); (ii) the peak position along the azimuthal angle χ, which reflects the in‐plane crystallographic orientation; and (iii) the full width at half maximum (FWHM) in Q, which can provide information about microstrain and strain heterogeneity within the probing area (Further discussion about the FWHM interpretation in the Supporting Information). Last, note that since in our XRD setup the *c*‐LP lies parallel to the incident beam, out‐of‐plane reflections defined by the *c*‐LP could not satisfy the Bragg condition in this configuration and were therefore not accessible.

Two complementary measurement regimes were used in this work. First, **macroscopic strain evolution** was studied using a larger beam size (103 × 87 $\mu m^{2}$), which measures over a relatively large sample region providing valuable averaged information on strain transfer (Figure 1e). Second, **spatially resolved strain mapping** was performed using a scanning XRD approach, where a focused beam (18 × 5 $\mu m^{2}$) was raster‐scanned across the membrane while recording diffraction patterns. By fitting the Bragg peaks at each position, we extracted maps of the in‐plane strain ($\varepsilon_{x}$, $\varepsilon_{y}$), FWHM, and orientation to study the local distribution of these parameters (Figure 1f). Throughout this work, we use the following terminology consistently: *Microstrain* refers to the FWHM of the Bragg peak extracted at each pixel in the spatially resolved maps, as broadening at this scale reflects mostly strain variations within the probed area – arising from cracks, wrinkles or local strain gradients – that cannot be spatially resolved at the pixel level. *Strain heterogeneity* refers to the spatial variation of the peak position – and hence of the local strain – between pixels across the map, reflecting macroscale heterogeneity in strain transfer over the membrane area. In the macroscopic measurement regime, the larger beam footprint integrates contributions from both effects simultaneously, so that their separation requires spatially resolved mapping.

### 2.1 Macroscopic Strain Evolution

**FIGURE 2:**
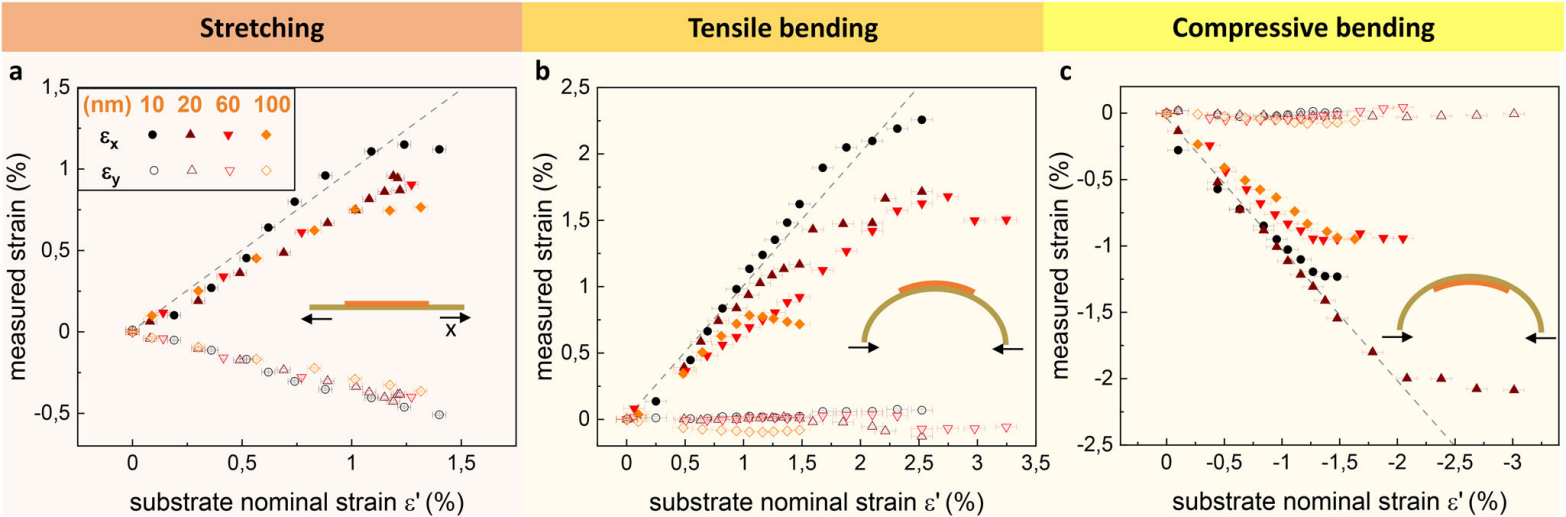
Mechanical stress mode effect on in‐plane strain. (a) Stretching, (b) tensile bending and (c) compressive bending uniaxial mechanical stress modes applied on LSMO membranes of 10, 20, 60, and 100 nm‐thick supported on a PI substrate. The resulting strain $\varepsilon_{x}$ (*a*‐LP, aligned with the stress direction) and $\varepsilon_{y}$ (*b*‐LP, in‐plane transverse to stress direction) are plotted as a function of the substrate nominal strain ($\varepsilon^{\prime }$).

First, we investigated the macroscopic strain evolution in LSMO membranes with different thicknesses (10, 20, 60, and 100 nm). Figure 2 shows the influence of the applied mechanical stress mode on the membrane strain $\varepsilon_{x}$ (aligned with the stress direction) and $\varepsilon_{y}$ (in‐plane transverse to stress direction).

A common trend, independent of thickness, is observed in the strain response along the stress direction: $\varepsilon_{x}$ increases under tensile stress (i.e. stretching, Figure 2a, and tensile bending, Figure 2b) and decreases under compressive bending stress (Figure 2c), as one would expect. In all cases, this evolution is initially linear up to a critical strain, beyond which the response collapses. A dashed line representing equal measured and applied strain (slope = 1) is included as a guide to the eye; deviations from this line mark the onset of the critical strain. This collapse most likely arises from crack formation within the membrane, as previously reported [21, 22, 43]. The mechanisms underlying this collapse, supported by optical microscopy, SEM, and diffraction peak width analysis, are discussed in detail below.

In contrast, strain variations in $\varepsilon_{y}$ (*b*‐LP of the LSMO membrane, in‐plane transverse to the stress direction) depend strongly on the stress mode. Under stretching, the $\varepsilon_{y}$ oppositely contracts, consistent with the Poisson effect of the flexible substrate: as the PI substrate elongates along the stress direction (= $\varepsilon_{x}$ direction), it contracts transversely, and this contraction is efficiently transferred to the membrane. Indeed, if we calculate the expected $\varepsilon_{y}$ using the Poisson's ratio of the PI substrate $\nu_{PI}[$ 44] ($\varepsilon_{y}=-\nu_{PI}\varepsilon_{x}$), we see that it closely matches the measured $\varepsilon_{y}$ in the linear regime (Figure S3). Under bending, however, the situation is fundamentally different. The deformation is accommodated by a strain gradient through the substrate thickness, with the outer and inner surfaces experiencing tensile and compressive strain, respectively, while the neutral plane at mid‐thickness remains unstrained and the total substrate volume unaltered. As a result, the overall transverse contraction is negligible, and the membrane clamped to the surface exhibits negligible transverse strain [45]. This contrast in $\varepsilon_{y}$ depending on the stress mode proves that the in‐plane strain symmetry in the membrane is governed by the in‐plane mechanical strain distribution within the supporting substrate. Although the out‐of‐plane *c*‐LP could not be measured directly in this configuration, Poisson's law predicts a dependence on the other two lattice parameters, and therefore it should depend on the stress mode as well. Indeed, calculations of the expected out‐of‐plane strain ($\varepsilon_{z}$) show that $\varepsilon_{z}$ compensates differently under stretching and bending due to the distinct behavior of $\varepsilon_{y}$ (Figure S4). Altogether, these findings demonstrate that the in‐plane strain of the membrane is dictated by the underlying flexible substrate and that the symmetry of strain in oxide membranes can be selectively tuned by the applied stress mode ‐ in this case bending vs stretching, using the same uniaxial device ‐ opening opportunities to access distinct physical responses [14, 16].

Examining the effect of thickness in Figure 2, strain transfer is more efficient in thinner membranes (10–20 nm) than in thicker ones (60–100 nm), which tend to collapse at lower applied stress. This behavior is consistent with the higher rigidity of thicker films, which also tend to contain more structural defects, making them more susceptible to cracking [21, 46]. For instance, comparing with other manganites, Hong et al. [14] showed that ultrathin 6 nm LCMO membranes could sustain uniaxial tensile strains up to 8% by stretching, whereas membranes thicker than 20 nm saturated below 2%. Our results follow the same trend overall, although in our case the maximum achievable strain by stretching is limited by the setup.

Having established how strain symmetry and transfer efficiency depend on stress mode and membrane thickness, we now discuss the mechanisms underlying the strain collapse in more detail using optical microscopy, SEM, and the evolution of the diffraction peak width. Optical microscopy of equivalent samples prepared under identical conditions and subjected to the same mechanical loading (Figure S5) confirms the interpretation of strain collapse: under tensile bending, cracks perpendicular to the loading direction nucleate at strains coinciding with the collapse in $\varepsilon_{x}$, while under compressive bending, buckling and wrinkling features ‐ also perpendicular to the loading direction ‐ appear from early applied strains and progressively evolve into cracking upon further loading, as observed by post‐mortem SEM analysis (Figure S6) [47, 48]. Furthermore, comparing the 20 and 60 nm thick membranes, cracks appear at lower applied strains in the thicker membrane under tensile bending, and the transition from buckling to cracking occurs at lower nominal strain under compressive bending, consistent with the earlier strain collapse and higher susceptibility to mechanical failure of the thicker membrane.

To further support the interpretation of the strain evolution and the onset of mechanical instability, we analyze the evolution of the FWHM of the diffraction peaks as a function of applied strain (Figure S7). While the strain data reveal a clear collapse beyond a critical value, the FWHM provides complementary information on the structural coherence of the film. An increase in FWHM is generally associated with enhanced structural disorder, mosaicity, or the coexistence of regions with slightly different lattice parameters. Importantly, due to the relatively large beam footprint used in these measurements, the extracted FWHM represents an average response over a significant portion of the membrane, and is therefore sensitive to heterogeneities that may develop during deformation. For the (200) reflection (stress direction), the FWHM generally remains relatively constant at low applied strain and increases sharply as the system approaches the critical strain, beyond which the strain collapse is observed. This behavior is consistent with the deformation domains identified in previous studies in brittle thin films on polymer substrates under in situ XRD loading: an initial plateau corresponding to elastic deformation with homogeneous strain, followed by pronounced broadening reflecting the onset of structural inhomogeneities associated with damage accumulation [49, 50]. On the other hand, the FWHM of the (020) reflection (transverse direction) does not exhibit significant systematic variation and remains relatively constant across different thicknesses and mechanical stress modes. This anisotropic broadening is further consistent with the microstructural observations from optical microscopy and SEM (Figures S3 and S4): cracks under tensile loading and buckled features under compressive loading are oriented perpendicular to the nominal strain direction, thereby disrupting $\varepsilon_{x}$ while leaving the transverse direction ($\varepsilon_{y}$) largely unaffected. This explains why the strain collapse and the sharp FWHM increase are observed exclusively in the (200) reflection, while the (020) reflection does not exhibit significant systematic variation.

Repeated bending tests (Figure S8a) revealed a slight reduction in measured strain for the same nominal substrate deformation after several cycles, and incomplete relaxation upon flattening, indicating the progressive formation of cracks that degrade strain transfer and recovery. The reversibility of the deformation depends critically on whether the applied strain remains below or exceeds the critical strain. When unloading from below the critical strain, the measured $\varepsilon_{x}$ returns to zero with negligible hysteresis, indicating elastic and reversible deformation (Figure S8b, top right inset). In contrast, when the substrate nominal strain exceeds the critical strain before unloading, the strain evolution exhibits a clear hysteresis loop and $\varepsilon_{x}$ after unloading becomes slightly tensile (Figure S6b, bottom left inset), consistent with permanent deformation of the membrane: buckled regions that formed irreversibly under compressive loading are left under a slight residual tensile stress once the substrate is flattened. Similarly, under tensile bending, cracks that form beyond the critical strain close upon unloading – rendering them invisible by optical microscopy (Figure S5) – but their presence is confirmed by SEM imaging of the unloaded samples (Figure S6), and their accumulation over repeated cycles accounts for the progressive reduction in strain transfer observed in Figure S8a. A similar hysteresis behavior upon unloading after reaching the critical strain was reported in indium tin oxide thin films on polymer substrates under tensile loading, where it was attributed to the progressive and asymmetric closing of cracks during unloading [51].

Beyond thickness and stress mode, several other factors also influence strain transfer efficiency including substrate composition and adhesion (Figure S9) [22], film quality, size and shape, and defects introduced during transfer. To assess reproducibility, we repeated the experiments on multiple membranes of identical thickness and observed small variations in the critical strain even among membranes from the same original sample (Figure S10). These results highlight that strain transfer in oxide membranes arises from a complex interplay of mechanical mode, film thickness, and several other factors, making it difficult to predict over extended strain ranges. Therefore, accurate in situ strain measurements are essential, as calculated nominal strain or ex situ measurements can overlook these effects.

To gain deeper insight into how strain distributes locally across the membranes and to disentangle the origin of the FWHM broadening, we next turn to spatially resolved strain mapping.

### 2.2 Spatially‐Resolved Strain Mapping

While macroscopic measurements provide valuable averaged information on strain transfer, they cannot capture local variations arising from defects, edges, or imperfect adhesion. To address this limitation and obtain a more complete picture of the spatial strain distribution, we performed HRXRD mapping. These measurements were carried out on the same samples used for the macroscopic strain evolution study at three representative nominal strain states ‐ pristine, low strain (within the linear elastic behavior), and high strain (close to mechanical failure)‐ enabling a direct correlation between macroscopic and local responses. Here, we focus on 10 nm‐thick (001) LSMO membranes subjected to (i) stretching and (ii) compressive bending stress, while additional measurements under other loading configurations and membrane thicknesses are provided in the Supporting Information.

#### 2.2.1 Stretching Mapping

**FIGURE 3:**
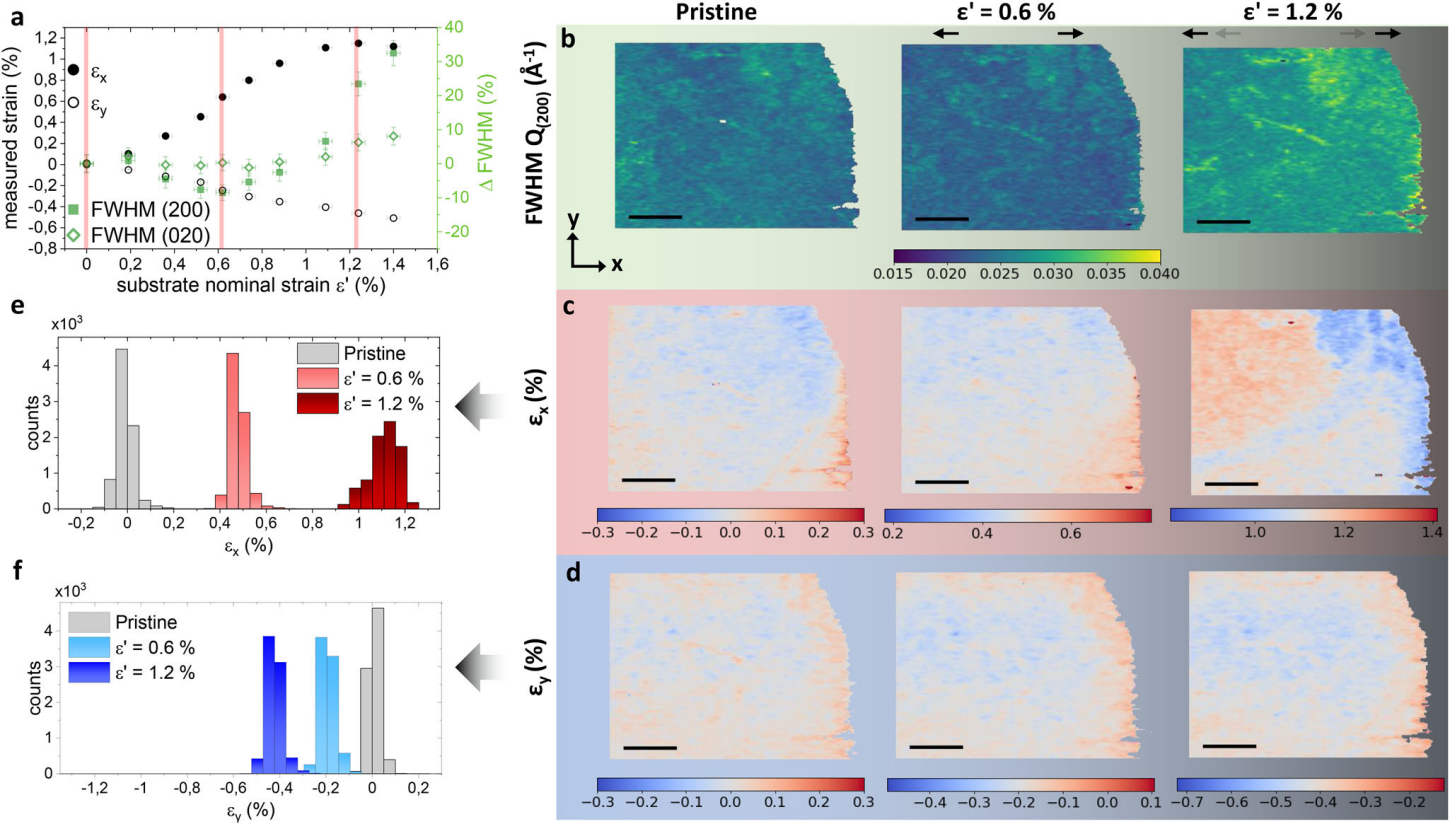
Spatially resolved analysis of a 10 nm LSMO membrane under stretching. (a) Macroscopic evolution of strain and FWHM broadening of the (200) and (020) reflections as a function of the applied nominal strain. The nominal strain values selected for spatial mapping are indicated by red lines. Maps are shown for (b) (200) FWHM in Q (Å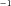), (c) strain in *a*‐LP ($\varepsilon_{x}$) and (d) strain in *b*‐LP ($\varepsilon_{y}$), each presented for the pristine state (left), $\varepsilon^{\prime }$ = 0.6 % (middle), and $\varepsilon^{\prime }$ = 1.2 % (right). The strain maps use a mean‐centered color scale with a fixed range of 0.6 %. The scale bar is 0.2 mm. The corresponding strain distributions extracted from the mappings are plotted for (e) $\varepsilon_{x}$ and (f) $\varepsilon_{y}$.

Figure 3 presents the spatially resolved strain mapping ($\varepsilon_{x}$, $\varepsilon_{y}$) and microstrain mapping (FWHM $Q_{\left(200\right)}$) of a 10 nm LSMO membrane on a PI substrate under stretching at three applied nominal strain states: $\varepsilon^{\prime }=0\%$ (pristine), $\varepsilon^{\prime }=0.6\%$ (linear regime), and $\varepsilon^{\prime }=1.2\%$ (strain collapse). Figure 3a shows the previously described macroscopic evolution, including the mean strain and the FWHM variation for the (200) and (020). The FWHM $Q_{\left(200\right)}$ (along the stress direction) first remains relatively constant, with a slight decrease at low applied strain ($\varepsilon^{\prime }∼0.6\%$) and then increases as the system approaches the critical strain ($\varepsilon^{\prime }>1\%$), while FWHM $Q_{\left(020\right)}$ (transverse direction) remains nearly constant. As previously described, possible sources of macroscopic FWHM variation include (i) microstrain (local lattice distortions like strain gradient, wrinkles, and defect‐related fluctuations, within the beam footprint) and (ii) macro‐scale strain heterogeneity.

To disentangle these effects on the macroscopic FWHM variations, we examined the spatially resolved FWHM maps together with the local strain distributions. Figure 3b shows the spatially resolved FWHM of the (200) reflection, which reflects local microstrain in the stress direction. In the pristine state, the microstrain is fairly homogeneous, with slightly larger values at defect‐rich regions. At low nominal strain ($\varepsilon^{\prime }=0.6\%$), the overall microstrain slightly decreases, consistent with the macroscopic FWHM trend. A plausible explanation is that mild tensile loading flattens wrinkles or surface corrugations introduced during release [35, 52], similarly to that observed for other 2D materials [53, 54]. This flattening reduces the strain gradients within the probing area, leading to a lower FWHM [55]. On the other hand, when the nominal strain increases up to $\varepsilon^{\prime }=1.2\%$, the FWHM rises sharply, indicating an increase in the microstrain. This high‐strain broadening likely reflects the accumulation of strain‐induced defects and the coexistence of strained and relaxed regions, consistent with the onset of the strain‐collapse regime [49, 50]. Since the crack spacing is smaller than the beam footprint (≈4 μm for 20 nm LSMO, Figure S6a), individual cracks cannot be spatially resolved in the maps but contribute collectively to the FWHM broadening. Nevertheless, the FWHM maps reveal spatial heterogeneity across the membrane, with larger values concentrated in specific regions likely associated with crack nucleation sites or areas of weaker adhesion.

Figure 3c,d shows the corresponding local strain maps for $\varepsilon_{x}$ and $\varepsilon_{y}$. Unlike the FWHM maps, these images reveal “macroscale” strain heterogeneity (> beam footprint). A consistent color scale (0.6% total range, centered at the mean strain value) highlights local deviations from the average: reddish areas correspond to higher‐than‐average strain and bluish to lower. Note that even the pristine membrane exhibits slight inhomogeneities in both $\varepsilon_{x}$ and $\varepsilon_{y}$, with a more compressive strain near the center than at the edges. This might originate from the residual compressive strain of LSMO when epitaxially grown on the ${\mathrm{SrCa}}_{2}{\mathrm{Al}}_{2}O_{6}$ sacrificial layer (lattice mismatch ≈ –0.2%) [24] which could be partially preserved during transfer with a polymeric stamp, while the edges relax more readily. Upon moderate stretching ($\varepsilon^{\prime }=0.6\%$), we observed that the mean strain increases without significant redistribution, which is consistent with the mean FWHM measured macroscopically. On the other hand, at higher loading ($\varepsilon^{\prime }=1.2\%$), strain variations clearly intensify in $\varepsilon_{x}$: regions near the edges and pre‐existing defects show reduced strain transfer (coinciding with zones of larger FWHM, Figure 3b), possibly indicating local relaxation through defects like microcrack formation. For $\varepsilon_{y}$, variations are smaller overall, but the same trend persists with lower strain transfer at edges compared to the membrane center. This relaxation at the edges could be explained by partial slippage of the membrane, a common issue for efficient strain transfer in supported 2D materials due to the weak van‐der‐Waals adhesion [30, 56, 57, 58].

Finally, the strain‐distribution histograms in Figure 3e–f complement the maps by showing the evolution of the local strain population. They confirm the overall macroscopic trend of increasing $\varepsilon_{x}$ and compensating $\varepsilon_{y}$, while also evidencing the broadening of the strain distribution with increasing applied stress. This statistical representation emphasizes that high substrate nominal strain not only changes the mean membrane strain value but also increases the underlying heterogeneity, consistent with the observed FWHM broadening and partial strain collapse at the macroscopic scale.

Similar experiments were carried out on thicker membranes of 60 nm (Figure S11) and 100 nm (Figure S12), which overall reproduce the trends described for the 10 nm case. In all cases, the macroscopic peak width remains nearly constant at low nominal strain and only starts to broaden significantly once the applied stress approaches the collapse regime, which correlates in the mappings with a small microstrain for low loadings which then becomes larger with increased strain heterogeneity as it gets closer to collapse. This behavior supports the interpretation that small tensile loads initially help to flatten the membrane, reducing local strain variations, whereas larger deformation introduces structural disorder and defect formation that increase the overall strain dispersion.

#### 2.2.2 Bending Mapping

**FIGURE 4:**
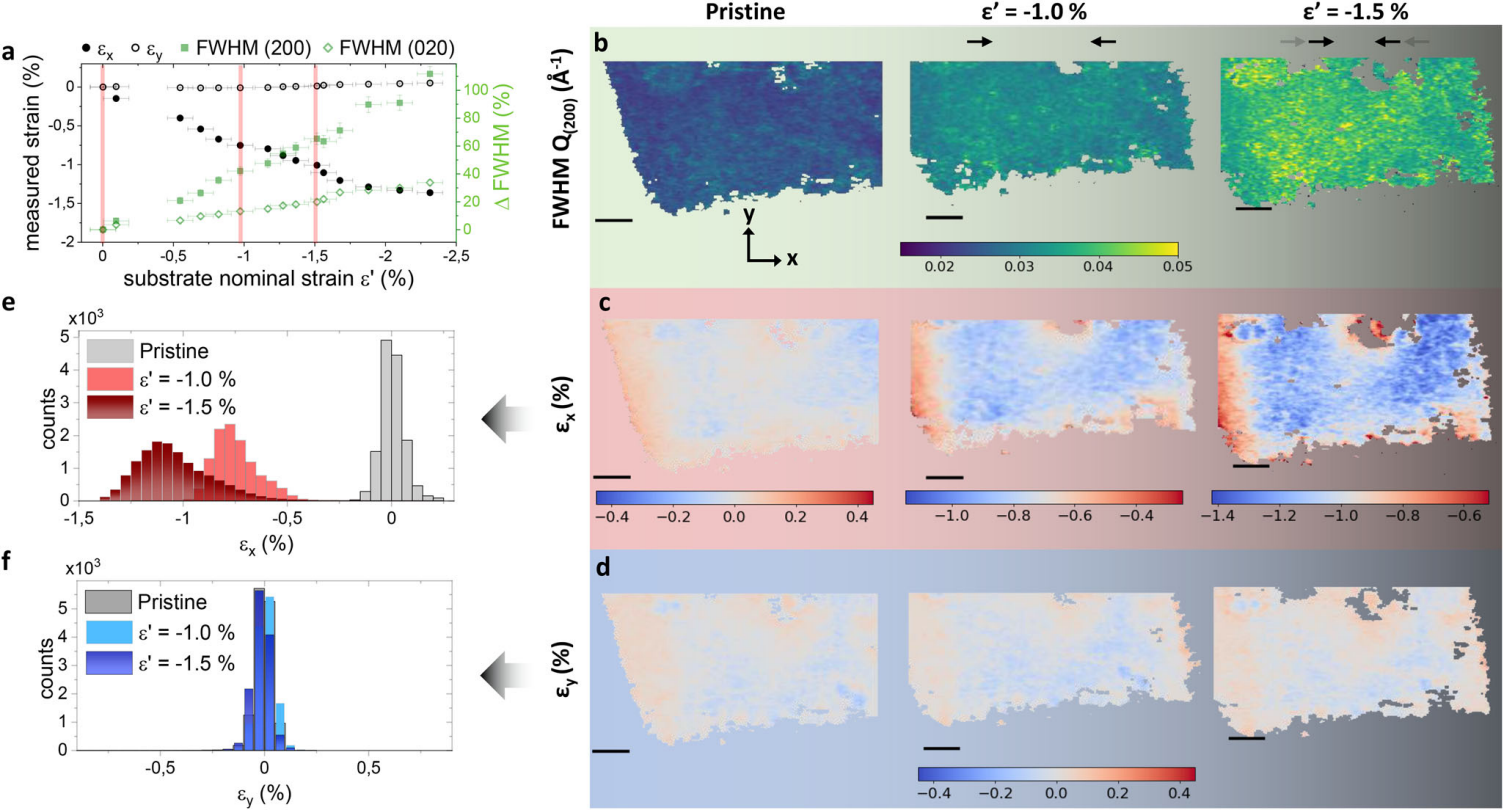
Spatially resolved analysis of a 10 nm LSMO membrane under compressive bending. (a) Macroscopic strain evolution and FWHM broadening of the (200) and (020) in‐plane reflections as a function of applied nominal strain. The nominal strain values selected for spatial mapping are indicated by red lines. Maps are shown for (b) (200) FWHM in Q (Å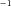), (c) strain in *a*‐LP ($\varepsilon_{x}$), and (d) strain in *b*‐LP ($\varepsilon_{y}$), each presented for the pristine state (left), $\varepsilon^{\prime }$ = –1.0 % (middle), and $\varepsilon^{\prime }$ = –1.5 % (right). The strain maps use a mean‐centered color scale with a fixed range of 0.9 %. The scale bar is 0.2 mm. The corresponding strain distributions extracted from the mappings are plotted for (e) $\varepsilon_{x}$ and (f) $\varepsilon_{y}$.

To compare the effect of stress mode on the spatial strain distribution, we performed a similar experiment on a 10 nm LSMO membrane, but now under bending mode rather than stretching. Figure 4 summarizes the results for compressive bending (see Figure S13 for the tensile case). Figure 4a shows the macroscopic strain evolution and corresponding macroscopic FWHM variations as a function of the applied nominal strain. As previously discussed, the macroscopic strain trend for bending differs from stretching, as in this case the transverse in‐plane strain ($\varepsilon_{y}$) remains essentially unaffected across the full strain range. Regarding the FWHM, it broadens with increasing compression, particularly along the stress direction, indicating a gradual buildup of microstrain and/or strain heterogeneity within the probed region.

To correlate these macroscopic observations with local strain variations, Figure 4b–d displays the spatially resolved FWHM of the (200) reflection and the strain maps ($\varepsilon_{x}$ and $\varepsilon_{y}$) for the pristine membrane, at low compressive strain ($\varepsilon^{\prime }=$– 1.0%, within the linear elastic regime) and at larger compression ($\varepsilon^{\prime }=$– 1.5%, approaching collapse). As shown in Figure 4b, the FWHM increases uniformly across the membrane as compression rises, consistent with a progressive accumulation of microstrain. This increase in microstrain is likely associated with the onset of wrinkling or buckle‐driven deformation of the membrane under compression [59]. This is consistent with the optical microscopy observations (Figure S5b), which show that buckling and wrinkling features appear from early applied compressive strains, well before the critical strain is reached. Since the lateral dimensions of these features (∼2 μm for 20 nm LSMO, Figure S6c) are smaller than the beam footprint, they cannot be resolved individually in the maps but contribute collectively to the progressive FWHM broadening. Similarly, early onset of FWHM increase driven by strain heterogeneities has been reported in tensely strained films where, in that case, interface waviness generated local strain variations that broaden the diffraction peak even in the nominally elastic regime [60].

At the same time, the spatial maps of $\varepsilon_{x}$ (Figure 4c) reveal a clear broadening of the strain distribution with increasing compression. In the pristine state, $\varepsilon_{x}$ shows slight compression at the membrane center and partial relaxation near the edges, resembling the pristine stretched sample previously discussed (Figure 3c). As the nominal strain increases to –1.0% and –1.5%, both the mean $\varepsilon_{x}$ and its spatial variability increase: edge and defect‐rich regions exhibit reduced strain transfer (reddish areas), and local strain differences up to $\Delta \varepsilon_{x}\approx 0.8\%$ emerge within the mapped area. This growing heterogeneity, evidenced by the strain distribution histogram (Figure 4e) and consistent with the increased microstrain (Figure 4b), likely originates from partial delamination or interfacial slippage between the membrane and the flexible substrate at the edges, triggered by buckling under compression [61]. Therefore, the macroscopic FWHM broadening observed in Figure 4a arises from two concurrent mechanisms: (i) a gradual increase in local microstrain and (ii) the development of larger‐scale strain heterogeneity across the membrane. Meanwhile, $\varepsilon_{y}$ remains close to zero at all deformation states (Figure 4d,f), confirming that bending does not induce a measurable transverse opposite strain, consistent with the absence of a Poisson‐like response.

### 2.3 Strain Heterogeneity and Its Impact on Functional Properties

**FIGURE 5:**
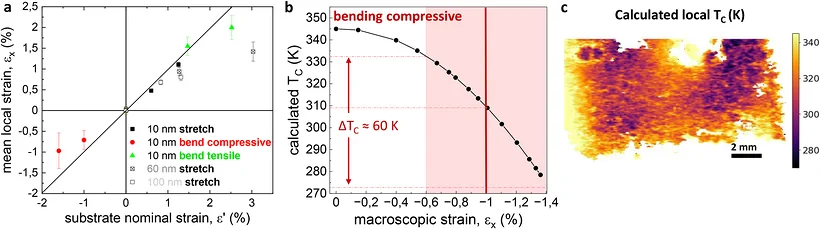
Correlation between strain heterogeneity and functional response in LSMO membranes. (a) Mean in‐plane strain $\varepsilon_{x}$ and standard deviation calculated from the spatially resolved strain mappings for 10, 60, and 100 nm LSMO membranes under different stress modes. (b) Curie temperature ($T_{C}$) calculated for a compressively bent 10 nm LSMO membrane. The red line represents the mean measured strain and the shaded area its standard deviation according to the local strain mapping under $\varepsilon^{\prime }=-1.5\%$; (c) shows the corresponding spatially resolved $T_{C}$ distribution.

To place these results in a broader context, Figure 5 first provides a statistical overview of the spatially resolved strain mapping data across all measured membranes and then links the observed strain heterogeneity to a representative functional property. Figure 5a shows the mean in‐plane strain ($\varepsilon_{x}$) extracted from the mappings, together with its corresponding standard deviation, as a function of the applied nominal strain. At low applied strain ($\varepsilon^{\prime }<1\%$), the mean measured strain follows a nearly linear relationship with the nominal strain, in agreement with the macroscopic measurements, and the standard deviation remains very small, indicating a highly uniform strain distribution within the elastic regime. As the applied load increases and the system approaches the critical strain, this linearity progressively breaks down and the standard deviation grows sharply, signaling a strong increase of strain heterogeneity. Notably, the strain heterogeneity is more pronounced under compressive than under tensile strain (standard deviation vs. mean strain shown in Figure S14). For instance, for 10 nm LSMO membranes at a nominal bending strain $|\varepsilon^{\prime }|\approx$1.5 %, the mean measured strain $\varepsilon_{x}$ reaches +1.5 ± 0.2 % for tensile bending and –1.0 ± 0.4 % for compressive bending. This asymmetry indicates that strain transfer is less efficient under compressive bending, likely associated with the onset of wrinkling/buckling and partial detachment from the substrate that locally relieve the imposed compression.

Such spatial strain heterogeneity is expected to have important consequences for the functional behavior of correlated oxides. Because strain is intimately coupled to electronic bandwidth and orbital occupancy, even subtle strain variations can strongly affect their functional properties. To illustrate this connection, we estimated the Curie temperature ($T_{C}$)–the onset of long‐range ferromagnetic order, highly sensitive to lattice distortions [62]–from the measured strain using an adapted version of the Millis et al. model [63] (see Supporting Information). We note that this model was originally derived for biaxially strained epitaxial films; its application here, combined with the use of a Poisson‐law estimate for $\varepsilon_{z}$ rather than a direct measurement, introduces significant uncertainties. The resulting $T_{C}$ values should therefore be regarded as a qualitative illustration of the expected trend rather than a quantitative prediction.

Applying this approach to the 10‐nm LSMO membrane under compressive bending, the calculated $T_{C}$ derived from the macroscopic strain evolution (Figure 5b) decreases from 345 K for the relaxed membrane to ∼280 K for $\varepsilon_{x}=-1.3\%$. This strong strain dependence of $T_{C}$ becomes particularly relevant when the strain is not spatially uniform. As an example, the large standard deviation (±0.4%) previously measured from the mappings for a mean $\varepsilon_{x}=$ –1.0 %, is represented in Figure 5b as a shaded red area, indicating that $T_{C}$ could vary by up to ∼60 K within the sample, as an illustrative estimate. These spatial variations are visualized in Figure 5c, where the local $T_{C}$ is calculated across the membrane, revealing regions where strain fluctuations lead to substantial deviations from the average $T_{C}$. Such inhomogeneity would result in coexisting ferromagnetic and paramagnetic regions at a given temperature, effectively broadening the magnetic transition and suggesting how spatial strain variations could locally tune the magnetic ground state in oxide membranes.

### 2.4 Crystallographic Orientation Mapping and Strain Transfer in Twisted Membrane Stacks

A key capability enabled by performing high‐energy x‐ray diffraction in transmission geometry on crystalline oriented oxide membranes, combined with a 2D detector, is the possibility to resolve and map the in‐plane crystallographic orientation with high angular sensitivity through the azimuthal angle χ (Figure 1d,f).

This configuration allows accessing local variations in the in‐plane orientation across the membrane surface, providing additional insight into how mechanical deformation affects not only lattice parameters but also subtle orientation changes. For instance, in a stretched 60 nm LSMO membrane (Figure S11e), local variations in χ correlate with strain redistribution within the membrane, revealing stress‐induced reorientation effects that are not captured by macroscopic averaging.

Beyond single membranes, such in‐plane orientation resolution naturally opens the opportunity to study stacked and twisted membrane architectures, which are of growing interest for multilayer heterostructures and oxide‐based twistronics [31]. In these systems, small relative rotations between layers can strongly modify interlayer coupling and strain transfer. For example, recent theoretical work predicts that stacked and twisted ${\mathrm{CoFe}}_{2}O_{4}$ membranes could enable reversible parallel–antiparallel magnetic domain switching under applied strain, emphasizing the importance of experimentally resolving strain and orientation at the individual‐layer level [33].

**FIGURE 6:**
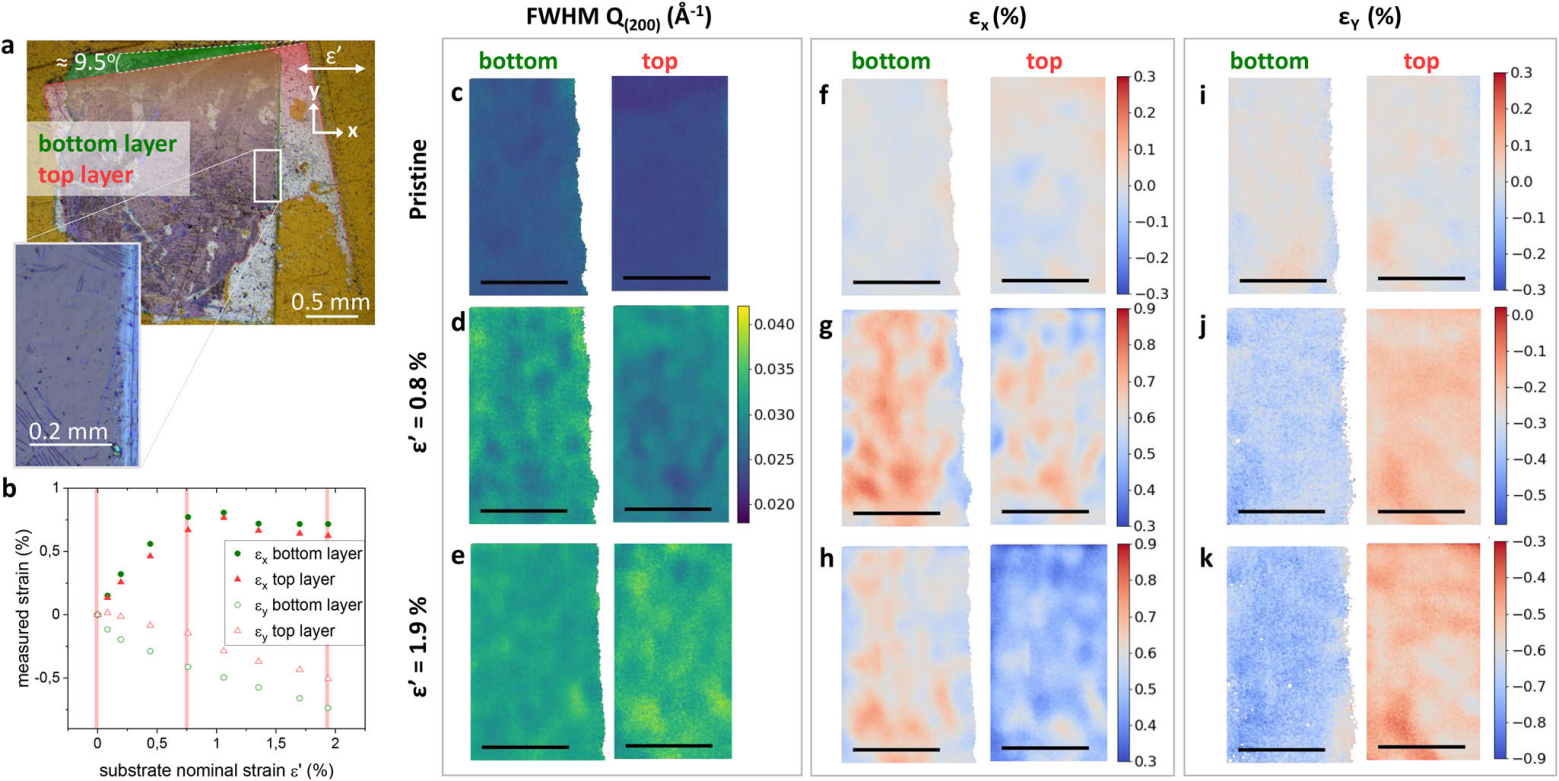
Strain mapping of two LSMO membranes stacked and twisted under stretching. (a) Optical microscopy image of two 20 nm LSMO membrane stacked and twisted $\approx 9.5^{\circ }$. The bottom and top layers are false‐colored in green and red, respectively. The white rectangle indicates the scanned area, shown as a magnified inset. The direction of applied strain is indicated. (b) Macroscopic strain evolution $\varepsilon_{x}$ and $\varepsilon_{y}$ as a function of applied substrate nominal strain $\varepsilon^{\prime }$, extracted independently for the bottom and top layers. (c–e) Spatially resolved maps of the FWHM of the (200) Bragg reflection (in Å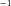) for the bottom and top layers in the pristine state (c), at $\varepsilon^{\prime }=0.8\%$ (d), and at $\varepsilon^{\prime }=1.9\%$ (e). (f–h) Corresponding maps of the in‐plane strain component $\varepsilon_{x}$ (%) for the bottom and top layers at the same three loading states. (i–k) Corresponding maps of the transverse in‐plane strain component $\varepsilon_{y}$ (%) for the bottom and top layers at the same three loading states. In all maps, the color scale is shared between bottom and top panels within each row, with a fixed FWHM range and a in‐plane strain range of 0.6 %. Scale bar in the mappings: 0.2 mm.

Figure 6 illustrates the capability of individually probing stacked membranes under applied mechanical loading using two 20 nm LSMO membranes on a PI substrate, stacked and twisted with a relative misorientation of $\approx 9.5^{\circ }$ in the azimuthal direction (χ) (optical image in Figure 6a). Owing to the use of a 2D detector, the diffraction contributions from each layer can be cleanly disentangled as their Bragg reflections are angularly separated and do not overlap.

Figure 6b shows the macroscopic evolution of the in‐plane strain components $\varepsilon_{x}$ and $\varepsilon_{y}$ for both layers as a function of the applied substrate nominal strain $\varepsilon^{\prime }$. Both layers follow the same qualitative trend and exhibit the same critical strain onset, confirming that mechanical loading is effectively transmitted through the stack. Quantitatively, however, the top layer consistently shows a reduced strain transfer: at $\varepsilon^{\prime }=0.8\%$, the average $\varepsilon_{x}$ is 0.77% for the bottom layer and 0.66% for the top layer, while $\varepsilon_{y}$ reaches −0.40% and −0.15%, respectively. This attenuation could indicate that the top layer is being partially screened from the substrate deformation by the intervening bottom membrane.

To spatially resolve the strain evolution of the stacked membranes, the maps of the FWHM of the (200) reflection (Figure 6c–e), $\varepsilon_{x}$ (Figure 6f–h) and $\varepsilon_{y}$ (Figure 6i–k) are extracted independently for each layer at three loading states: pristine, $\varepsilon^{\prime }=0.8\%$ (approaching the critical strain), and $\varepsilon^{\prime }=1.9\%$ (above the critical strain), where the critical strain onset is reached at approximately $\varepsilon^{\prime }\approx 0.9\%$. The FWHM maps (Figure 6c–e) show a progressive increase with applied strain for both layers, with no significant differences between the bottom and top layers in either magnitude or spatial distribution. This overall increasing microstrain is consistent with the onset of cracking when approaching the critical strain due to the unresolved crack features within a pixel (beam footprint). The $\varepsilon_{x}$ and $\varepsilon_{y}$ maps (Figure 6f–k) show that strain transfer to the top layer is consistently reduced relative to the bottom layer across all loading states. This attenuated strain transfer to the top membrane could be partly attributed to the imperfect interlayer contact inherent to non‐epitaxially stacked membrane architectures: cross‐sectional electron microscopy studies have revealed the presence of a nanometric gap between membranes after transfer, arising from surface contamination introduced during the wet etching process [64]. Further processing steps such as thermal annealing – shown to reduce gap width and promote interfacial bonding – could provide a route to improving interlayer strain coupling in stacked architectures. Notably, despite this attenuation, the spatial distribution of strain is strikingly correlated between layers: regions of higher strain in the bottom membrane tend to coincide with regions of higher strain in the top membrane (Figure 6g,h), suggesting that local strain heterogeneities are transmitted across the interface in a spatially coherent manner. This difference in strain magnitude between the two layers becomes more pronounced above the critical strain ($\varepsilon^{\prime }=1.9\%$, Figure 6h,k), which may be related to cracking‐induced relaxation of the bottom layer, partially disrupting the interlayer contact and further reducing strain transfer to the top membrane.

Altogether, this approach provides a powerful route to investigate how strain is transferred, redistributed, and attenuated across layers in multilayer oxide membrane architectures, with layer‐resolved spatial resolution that macroscopic averaging techniques cannot provide.

## 3 Conclusions

In summary, we have unveiled how strain is transferred and distributed in oriented oxide membranes under external mechanical loading, providing a comprehensive picture of strain engineering in these systems. These findings were made possible by applying synchrotron HRXRD with spatially resolved strain mapping which enables quantitative, in situ investigation of strain at both macroscopic and micrometer scales.

At the macroscopic level, we find that the efficiency of strain transfer depends critically on the membrane thickness and supporting substrate, while the symmetry of strain is dictated by the applied stress mode. Thinner membranes (10, 20 nm) transfer strain more efficiently than thicker ones (60, 100 nm), which are more rigid and prone to cracking. The strain symmetry is governed by the clamping to the flexible substrate, producing distinct responses for stretching and bending: stretching leads to transverse contraction, whereas bending results in a quasi‐uniaxial in‐plane strain state. In all cases, the transferred strain collapses beyond a critical strain, reflecting the onset of crack formation and strain relaxation, consistent with the observed broadening of the diffraction peaks studied.

Spatially resolved strain mapping provides deeper insight into these behaviors, allowing us to disentangle two complementary contributions to the overall peak broadening: microstrain at the local scale and strain heterogeneity across larger regions of the membrane. While local microstrain generally increases with the applied stress, its evolution differs between tensile and compressive loading at low strain, likely due to the wrinkling/buckling under compression. On the other hand, strain heterogeneity increases systematically with applied stress, originating primarily from the edges and defect‐rich regions, where strain transfer is less efficient. To illustrate the potential functional implications, we estimate local variations in the Curie temperature arising from spatial strain inhomogeneity, suggesting possible shifts of several tens of kelvin within a single membrane. Furthermore, the present approach enables the independent, layer‐resolved strain characterization of stacked and twisted oxide membrane architectures, as demonstrated here for a twisted bilayer stack under stretching, where strain transfer is found effective yet partially attenuated with increasing distance from the substrate.

Beyond these findings, this work underscores the importance of measuring strain in oxide membranes with high accuracy, spatial resolution, and in situ capability. Because strain transfer in these systems is inherently complex and sensitive to multiple factors – including thickness, adhesion, and stress mode – only such comprehensive characterization can capture the true strain distribution and its evolution under external loading. Accurately resolving these effects is therefore essential to understand and predict how local structural distortions impact the functional response of oxide‐based devices.

Moreover, these capabilities open exciting opportunities for operando investigations, enabling direct correlation between mechanical deformation and functional performance under realistic operating conditions, as well as for the design of stacked and twisted oxide membrane heterostructures where interlayer coupling and rotational alignment could further modulate strain and electronic behavior.

## 4 Methods

### 4.1 LSMO Membranes Preparation

The LSMO freestanding membranes were fabricated using a sacrificial‐layer water‐etching approach, which allows epitaxial oxide films to be released from their rigid substrates. Epitaxial LSMO films were first grown on single‐crystalline (001) ${\mathrm{SrTiO}}_{3}$ (STO) substrates coated with a water‐soluble sacrificial layer. Two different heterostructure configurations were used: (i) LSMO grown by pulsed laser deposition (PLD) on a chemically solution–deposited ${\mathrm{SrCa}}_{2}{\mathrm{Al}}_{2}O_{6}$ sacrificial layer [24], and (ii) PLD‐grown LSMO on a PLD‐grown ${\mathrm{Sr}}_{3}{\mathrm{Al}}_{2}O_{6}$ sacrificial layer [22]. After growth, the samples were coated with a polypropylene carbonate (PPC) stamp prior to immersion in deionized water to selectively dissolve the sacrificial layer. The released membranes were subsequently transferred using the PPC stamp onto flexible polymer substrates – 50 μm thick polyimide (PI) sheets [44] for the main study, but also alternative substrates were explored in control experiments. Once transferred, the PPC was removed by mild heating at 100
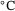
–120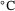 for 30 min followed by acetone rinsing, leaving the LSMO membrane cleanly adhered to the underlying substrate and ready for mechanical testing and synchrotron characterization. Laboratory‐scale XRD characterization of the as‐grown heterostructures and the released membranes prior to synchrotron measurements, confirming phase purity and strain relaxation upon lift‐off, is provided in the Supporting Information (Figure S15). For the stacked membrane configuration, a second LSMO membrane was transferred first onto a PET/silicone stamp and then onto a previously deposited membrane on PI by pressing at 90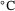 for 30 min, after which the PET/silicone support was released, yielding a two‐layer stack adhered to the PI substrate.

### 4.2 HRXRD Synchrotron Measurements

The synchrotron High Resolution X‐Ray Diffraction (HRXRD) measurements were performed on the ID31 beamline at the European Synchrotron Radiation Facility (ESRF‐EBS, Grenoble, France) using a monochromatic 75.0 keV x‐ray beam. A custom‐built strain stage integrated into the diffraction setup was used to apply mechanical stress in situ to the sample while measuring strain. The membranes, supported on flexible polymer substrates, were clamped from two sides with two motor‐controlled arms used to apply uniaxial strain. By increasing the distance between the clamps, the substrate was stretched, while decreasing the distance induced bending deformation, which could be either compressive or tensile depending on the face of the flexible polymer substrate where the membrane was placed. A high‐precision goniometer was used to align the sample and to allow θ rotation during acquisition. A top‐view camera was employed to (i) maintain a fixed sample‐to‐detector distance and (ii) to calculate the nominal strain applied, either from the bending radius or the stretching displacement (Figure S1). The incident beam size could be tuned with the transfocators of the beamline from sub‐micron dimensions up to 400 × 400 $\mu m^{2}$, enabling measurements that probe either highly localized regions or large averaged areas across the membrane. Diffraction data were collected in transmission mode using a PilatuS3 CdTe 2M detector and calibrated with a ${\mathrm{CeO}}_{2}$ reference standard. The raw 2D diffraction images were converted into reciprocal‐space (χ–Q) maps using the beamline calibration parameters. Individual in‐plane Bragg reflections, such as (200) and (020), were then isolated and fitted with Gaussian profiles to extract quantitative information. From the peak position in Q we determined the in‐plane lattice parameters (*a*‐LP and *b*‐LP) and the corresponding strain components ($\varepsilon_{x}$ and $\varepsilon_{y}$), while the FWHM in Q provided the microstrain (Further discussion about the FWHM interpretation in the Supporting Information). The reported strain values are defined by $\varepsilon_{x}$ = Δa/$a_{0}$ where $a_{0}$ is the average lattice parameter of the pristine membrane (ε′ = 0) measured with a defocused beam (∼400 × 400 $\mu m^{2}$). Error bars on the measured lattice strain were determined by propagating the uncertainty on the Gaussian peak position fitted in Q. On the other hand, the peak position in χ yielded the crystal in‐plane orientation (Figure S2).

#### 4.2.1 Macroscopic Strain Evolution

For macroscopic strain evolution measurements, the sample was mounted on a goniometer and the mechanical load was applied stepwise using the strain stage. At each applied load, a hold period was maintained while the diffraction data were acquired. During each hold period, the sample was rotated in θ (Figure 1a) while continuously recording diffraction patterns, repeating the same procedure for each applied mechanical load. This procedure, analogous to a rocking curve scan, ensured that the Bragg reflection was always properly aligned with the incident beam, minimizing potential artifacts arising from slight misalignment or misorientation of the film relative to the incident beam induced by bending of the polymeric substrate. The final diffraction profile was reconstructed by summing the images acquired at different θ positions close to the peak maximum (± 3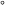), which improved resolution and accuracy in determining peak position. These measurements were done with a beam size of 103 × 87 $\mu m^{2}$ (H x V) in the center of the sample. Each strain evolution curve presented in this work corresponds to a single membrane, and sample‐to‐sample variability is assessed in Figure S10.

#### 4.2.2 Spatially Resolved Strain Mapping

Spatially resolved strain mapping was carried out using a scanning XRD approach, in which a focused beam was raster‐scanned across the sample while collecting a diffraction pattern at each position. Performing a full rocking curve at every pixel would have been prohibitively time‐consuming; therefore, an initial rocking curve was first recorded with a defocused beam (∼400 × 400 $\mu m^{2}$) to determine the average θ corresponding to the maximum intensity of the Bragg peak of interest. For flat samples, the subsequent mapping was carried out at this fixed θ value, assuming that the sample remained aligned at this orientation across the scanned area. For bent samples, the sample translation in *x* was synchronized with a rotation in θ based on the substrate bending radius, ensuring that the beam remained aligned with the Bragg peak of interest throughout the scan. Mappings were performed using a focused beam (18 × 5 $\mu m^{2}$), with the step size chosen according to the beam footprint to ensure appropriate spatial sampling. The acquisition time per point ranged from 0.05 to 0.1 s depending on the membrane's diffraction signal intensity.

## Conflicts of Interest

The authors declare no conflicts of interest.

## Supporting information


**Supporting File**: smll74801‐sup‐0001‐SuppMat.pdf.

## Data Availability

Raw X‐ray data generated at the ESRF large‐scale facility are available at https://doi.esrf.fr/10.15151/ESRF‐ES‐2045346549 and https://doi.esrf.fr/10.15151/ESRF‐ES‐2409235866 from 2028 and 2029, respectively.
