# Supplementary material for: Spatially Resolved Strain Mapping In Flexible Oxide Membranes
Source: Small. 2026 Jul 23;22(53):e74801. doi: 10.1002/smll.74801 (PMC13592415; doi:10.1002/smll.74801)
Supplement: Supplementary file 1 [file SMLL-22-e74801-s001.pdf]

Supporting Information for *Spatially resolved strain mapping in flexible oxide membranes*

Pol Salles,[*,†,‡,¶] Marti Ramis,[‡] Eric Brand,[§] Peter Kúš,[‖] Julie Ruellou,[†] Andrea Sartori,[†,⊥] José Manuel Caicedo Roque,[#] Dae-Sung Park,[§] Nini Pryds,[§] Mariona Coll,[‡] and Jakub Drnec[*,†]

†*ESRF, The European Synchrotron, 71 Avenue des Martyrs, CS40220, 38043 Grenoble Cedex 9, France*

‡*Institut de Ciència de Materials de Barcelona (ICMAB-CSIC), Carrer dels Til.lers, 08193 Cerdanyola del Vallès, Spain*

¶*Department of Mining, Industrial and ICT Engineering, Universitat Politècnica de Catalunya (UPC), Av. De les Bases de Manresa, 61-73, 08242 Manresa, Spain*

§*Department of Energy Storage and Conversion, Technical University of Denmark, 2800 Kongens Lyngby, Denmark*

‖*Department of Surface and Plasma Science, Faculty of Mathematics and Physics, Charles University, V Holešovičkách 2, Prague 8, 18000 Czech Republic*

⊥*Present address: ALBA Synchrotron, Spain*

#*Catalan Institute of Nanoscience and Nanotechnology (ICN2), CSIC and BIST, Campus UAB, Bellaterra, Spain*

E-mail: pol.salles@upc.edu; drnec@esrf.fr

Setup and data treatment

Determination of substrate nominal strain. As illustrated in Figure S1, the nominal strain applied to the substrate was determined from optical images acquired using a high-resolution camera (Basler AG) positioned above the sample. The images were processed to quantify the substrate nominal strain, ε', either from the measured elongation during stretching experiments or from the bending radius during bending experiments.

For stretching, the nominal strain was calculated as $\varepsilon' = \Delta L/L$, where L is the initial distance between two visible reference points on the substrate and ΔL is the corresponding change in distance upon deformation. The uncertainty in the applied strain was determined by repeating each distance measurement four times with independently selected reference point pairs; the resulting mean standard deviation is ± 0.03 %, which is used as a uniform error bar in all figures.

For bending experiments, the nominal strain was estimated as $\varepsilon' = t/(2r)$, where t is the substrate thickness and r is the bending radius extracted from the curvature of the substrate.[1] The uncertainty in the applied bending strain was determined for one representative sample by repeating the circle fit four times per image, each time manually selecting a different section of the substrate arc. The mean standard deviation across all bending positions is ± 0.09% which is applied as a uniform error bar for all samples and figures.

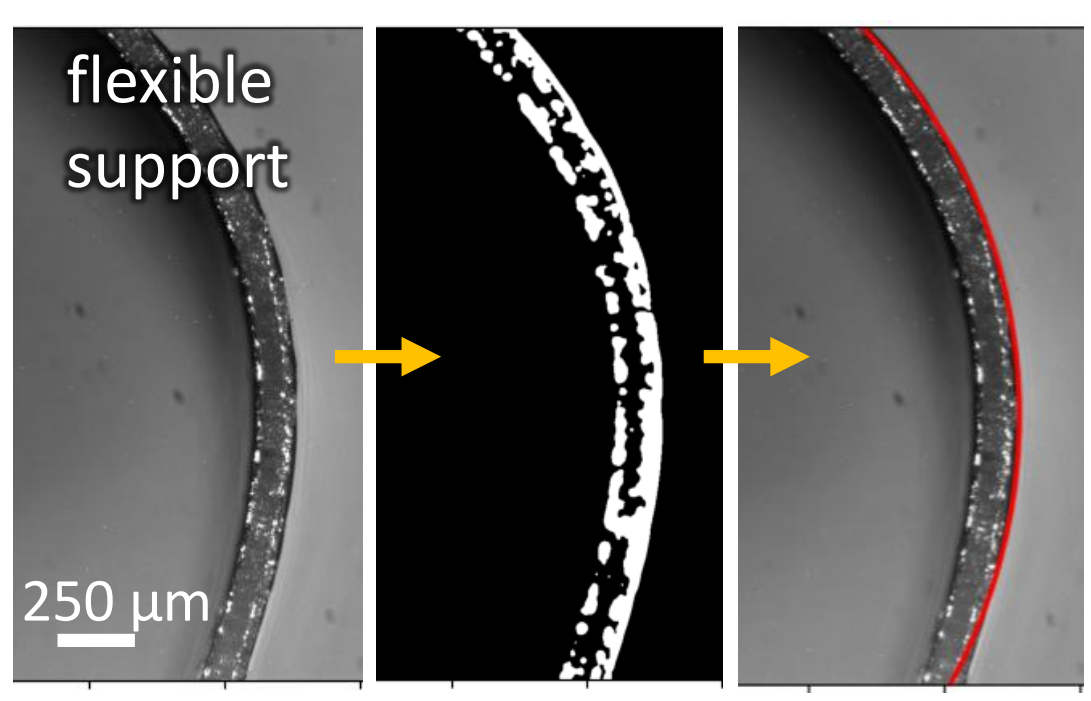

Figure S1: Procedure for determining the substrate nominal strain. Example shown for bending: (left) optical image of the bent substrate acquired with the top-view camera; (center) processed image used to extract the bending radius; (right) red line indicating the fitted curve used to calculate the bending radius.

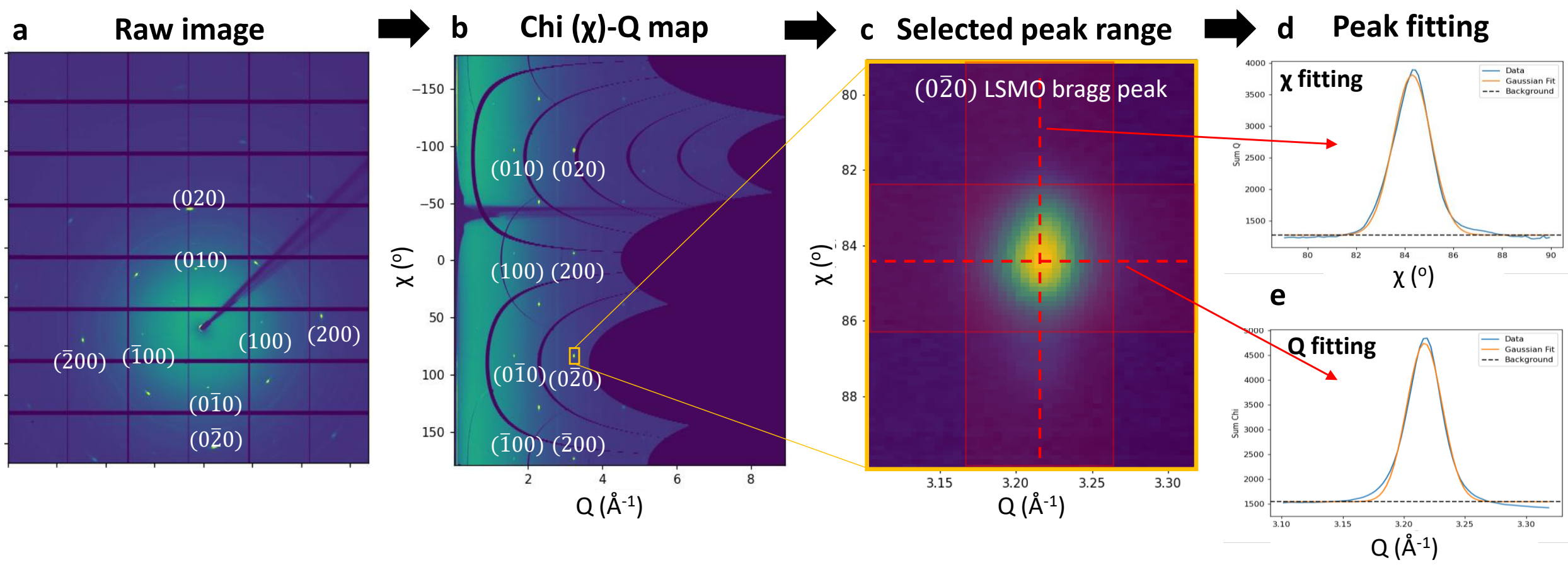

Figure S2: Workflow for extracting strain and orientation from HRXRD synchrotron data. (a) Raw 2D diffraction image is transformed into (b) a χ–Q map, where (c) individual Bragg peaks ((200) and (020)) are isolated to fit the a-LP and b-LP, respectively. (d,e) Gaussian fitting along the χ and Q directions yields peak positions and corresponding FWHM.

Macroscopic strain evolution

Poisson effect in ε_y predicted from substrate. For the stretched samples, the measured ε_y^{LSMO} is compared with the values calculated from the PI substrate's Poisson ratio ($\nu_{PI} = 0.34$)[2] by $\varepsilon_y^{LSMO} \approx \varepsilon_y^{PI} = -\nu_{PI} \cdot \varepsilon_x$. As shown in Figure S3, the calculated and measured curves closely match in the linear elastic regime, confirming that the transverse strain ε_y^{LSMO} of the membrane is governed by the substrate (i.e. the film follows the substrate's in-plane deformation and remains fully bonded). The main deviations occur at larger nominal strains, where the measured strain starts collapsing due to cracking, thereby breaking the linear elastic response.

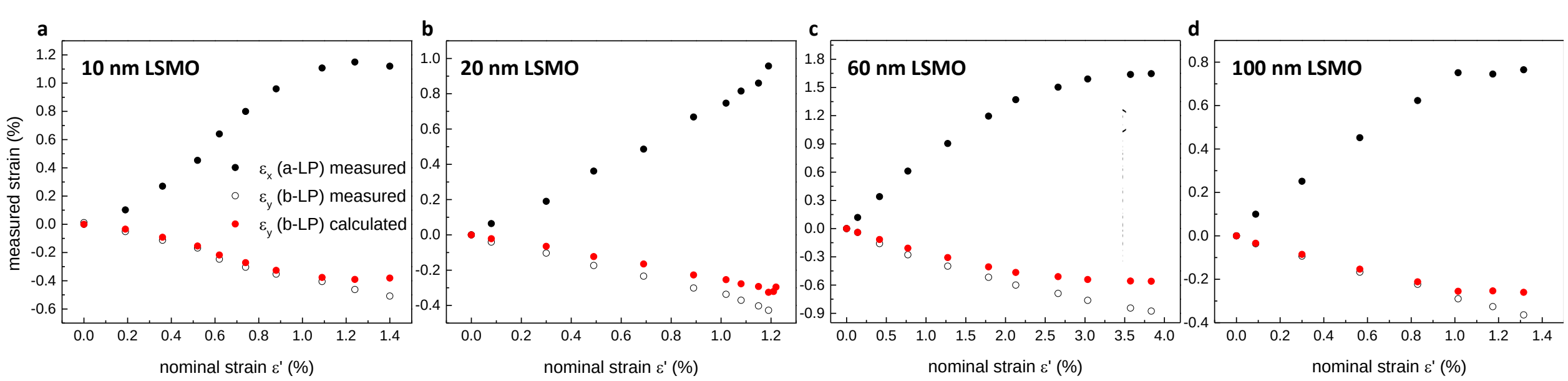

Figure S3: Macroscopic strain evolution in stretched LSMO membranes: measured versus calculated ε_y strain using Poisson's law of the substrate

Poisson effect in c-LP. Although the out-of-plane strain (ε_z, corresponding to the c-LP)

cannot be directly measured with the present experimental configuration, its expected value can be calculated from linear elasticity using Hooke's law. Assuming the membrane can be approximated as isotropic, that it remains in the linear elastic regime, and that the membrane surface is free so the out-of-plane stress condition ($\sigma_z = 0$) applies, the out-of-plane strain is

$$\varepsilon_z = \frac{1}{E}[\sigma_z - \nu(\sigma_x + \sigma_y)] = -\frac{\nu}{E}(\sigma_x + \sigma_y) = -\frac{\nu}{E}\left[\frac{E}{1-\nu}(\varepsilon_x + \varepsilon_y)\right] = -\frac{\nu}{1-\nu}(\varepsilon_x + \varepsilon_y) \quad (1)$$

where ν is the LSMO Poisson ratio ($\nu_{LSMO} = 0.37$)[3] and $\varepsilon_x, \varepsilon_y$ are the in-plane strains.

Figure S4 shows the calculated out-of-plane strain ε_z for stretched LSMO membranes of different thicknesses (Fig. S4a–d), as well as for 10 nm LSMO membranes subjected to mechanical bending in tensile (Fig. S4e) and compressive (Fig. S4f). In all configurations, the out-of-plane strain ε_z has the opposite sign to the applied in-plane strain ε', reflecting the Poisson response of the membrane, which tends to compensate changes in the LSMO volume. This behaviour is independent of thickness, as seen in the stretched samples (Fig. S4a–d). On the other hand, the magnitude of ε_z depends strongly on the stress mode. Comparing 10 nm membranes under stretching and bending (Fig. S4a,e,f), the calculated ε_z variation is larger under bending than under stretching. This trend is clearly seen when ε_z is compared for the different stress modes under a $|\varepsilon_x| = 1$ %, see Fig. S4g. Such difference in the calculated out-of-plane strain ε_z arises because, during bending, the transverse in-plane component ε_y is constrained by the polymer substrate ($\varepsilon_y \approx 0$). As a result, ε_z must accommodate most of the Poisson compensation of ε_x.

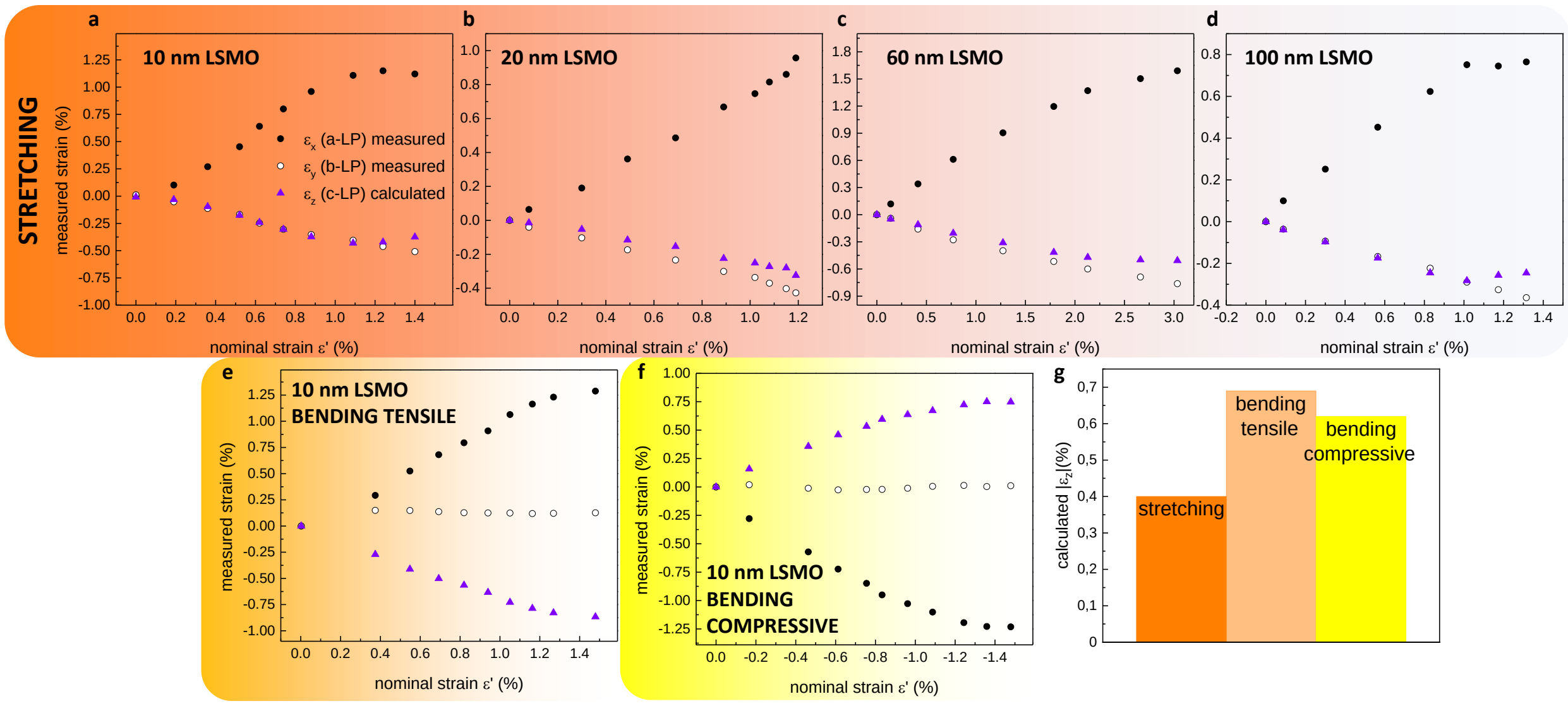

Figure S4: Calculated out-of-plane strain (ε_z) of LSMO membranes using Poisson's law. Results are shown for stretched membranes of thickness (a) 10 nm, (b) 20 nm, (c) 60 nm, and (d) 100 nm; (e) 10 nm membrane under tensile bending and (f) 10 nm membrane under compressive bending. (g) Calculated ε_z at $|\varepsilon_x| = 1$ % for 10 nm LSMO membranes subjected to the different stress modes.

Surface morphology characterization.

Optical images of the LSMO membranes supported on PI substrates were acquired during in situ bending using a Leica DM2700M optical microscope. Post-mortem surface morphology of the membranes after mechanical loading was examined by a SEM QUANTA FEI 200 FEG-ESEM.

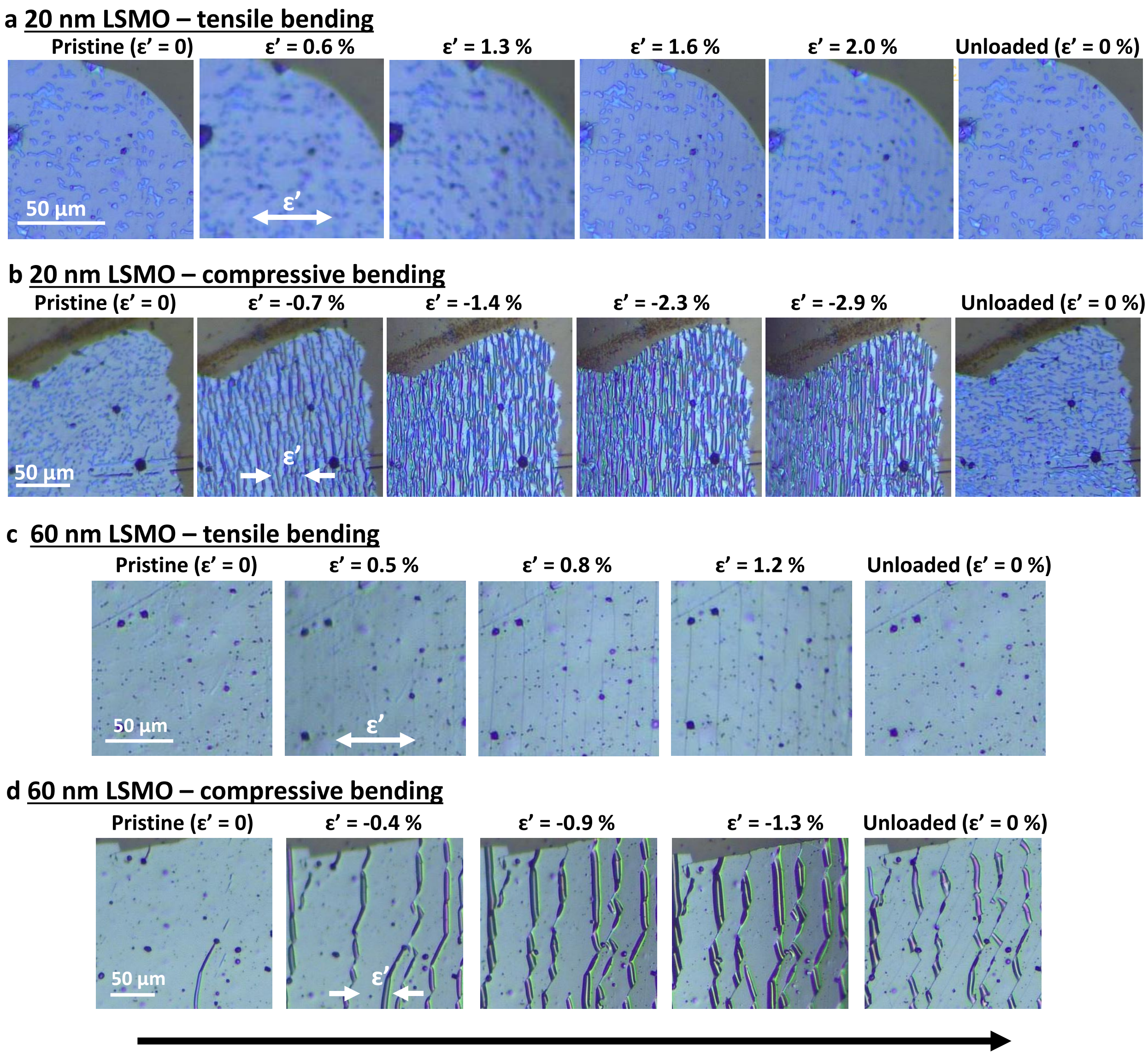

Figure S5: In situ optical microscopy of LSMO membranes under progressive mechanical bending. Sequential optical microscopy images acquired during (a,c) tensile bending and (b,d) compressive bending of 20 nm (a,b) and 60 nm (c,d) thick LSMO membranes on PI substrates. The loading direction (ε') is indicated with white arrows. The sequence direction that is followed is indicated by the bottom black arrow. Scale bar: 50 μm.

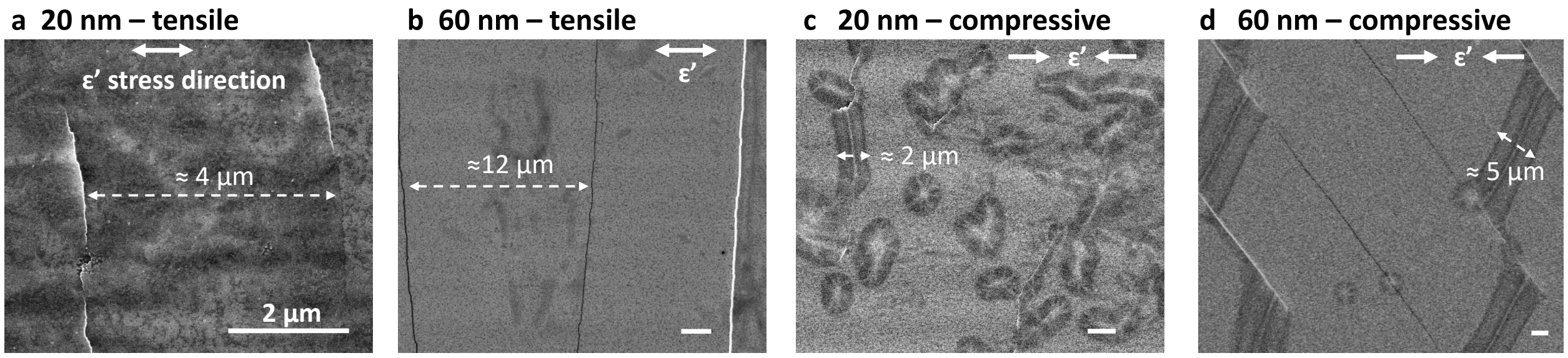

Figure S6: Post-mortem SEM images of LSMO membranes after bending. SEM images of (a,b) tensile-bent and (c,d) compressively-bent LSMO membranes of 20 nm (a,c) and 60 nm (b,d) thickness, after in situ optical microscopy and unloading in the flat configuration. The loading direction (ε') is indicated by arrows. Dashed arrows indicate the approximate spacing between cracks for tensile bending, and buckled characteristic lateral dimensions for compressive bending. Scale bar: 2 µm.

FWHM evolution under mechanical loading.

The full width at half maximum (FWHM) of a Bragg reflection measured in XRD can in principle receive contributions from several sources: (i) instrumental broadening, (ii) finite crystallite size (Scherrer broadening), (iii) mapping artifacts from fixed-angle acquisition, (v) sample curvature, mosaicity, and local tilt distribution and (vi) microstrain and strain heterogeneity within the beam footprint. Here we discuss each contribution and argue that the observed evolution of the FWHM under mechanical loading is dominated by the latter.

Instrumental broadening. The monochromator energy resolution $\Delta E/E = 8 \times 10^{-4}$ corresponds to a Q broadening approximately one order of magnitude smaller than the measured FWHM values, and is constant across all measurements. The cross talk between the pixels and signal spread over multiple pixels is negligible given our detector position. The analyzed peak spreads over 10 pixels and the actual artificial spread from one pixel to the other is effectively zero for photon counting detectors.

Finite size broadening. We assume no significant grain size change according to previous work on supported or freestanding single-crystalline oxide membranes under an applied loading. Above the critical strain, cracking produces fragment spacings of 4–12 μm, yielding a Scherrer broadening of $\Delta Q = 2\pi K/L \approx 1.4 \times 10^{-4}$ Å^{-1} for $L = 4$ μm, two orders of magnitude below the measured FWHM range of 0.01–0.04 Å^{-1}, and therefore does not contribute meaningfully to the observed evolution.

Fixed-angle mapping artifacts. Local membrane misalignment relative to the fixed diffraction angle could induce apparent broadening in specific regions. However, the FWHM evolution is spatially consistent across the maps and follows the same trends as the macroscopic measurements, ruling out mapping artifacts as a dominant contribution.

Sample curvature, mosaicity, and local tilt distribution. Macroscopic curvature in the bending geometry could broaden the peak if different regions within the beam footprint diffract at slightly different angles. Additionally, local structural features such as buckles and wrinkles — observed under compressive loading — introduce local membrane tilts that are physically coupled to strain gradients in their vicinity, both of which contribute to broadening in Q. However, we can exclude these effects as dominant contributions by comparing the FWHM evolution across stress modes: under stretching, where the substrate remains flat and no macroscopic curvature or buckling is present, the FWHM evolution is qualitatively comparable to that observed under bending. This consistency across geometrically distinct loading modes indicates that the observed broadening primarily reflects strain-related structural changes. We nonetheless acknowledge that buckling under compression may introduce a non-negligible secondary contribution to the FWHM in that specific regime, which cannot be fully disentangled from the strain gradient contribution at the current spatial resolution.

Microstrain and strain heterogeneity. Having argued that the above contributions are not dominant, we conclude that the FWHM increase with mechanical loading is primarily driven by two strain-related effects. In the macroscopic measurements (Figure S7), the beam footprint covers a large fraction of the membrane, so the FWHM integrates contributions from the full spatial **strain heterogeneity of the membrane**, including regions of different local strain, defects, edges, and damage features. In the spatially resolved maps, each pixel probes a smaller area (18 x 5 μm^2), so the local FWHM reflects **microstrain** within that pixel, including sub-pixel structural features such as incipient cracks or buckles, whose lateral dimensions are smaller than the beam footprint. These two contributions can be partially disentangled by comparing the pixel-level FWHM with the pixel-to-pixel variation of the peak position across the map, as discussed in the main text.

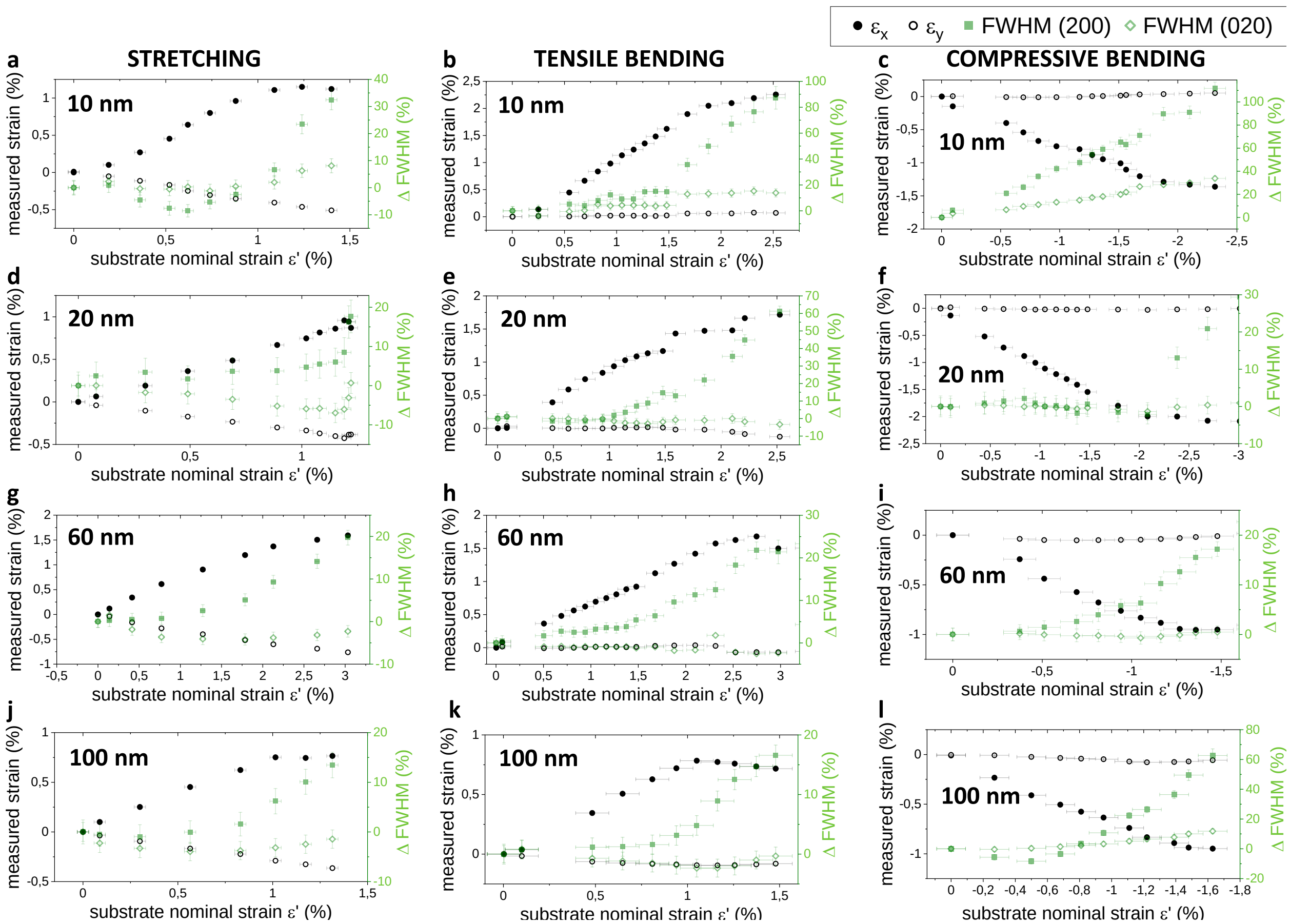

Figure S7: Macroscopic strain and FWHM evolution. The evolution of HRXRD strain ε_x and ε_y and the increment of (200) and (020) FWHM are presented for LSMO membranes of different thicknesses on PI subjected to different stress modes: 10 nm LSMO under (a) stretching, (b) tensile bending and (c) compressive bending; 20 nm (d-f), 60 nm (g-i) and 100 nm (j-l).

Membrane recovery. Figure S8a illustrates the effect of repeated tensile bending on strain transfer. A 10 nm LSMO membrane was subjected to five consecutive bending cycles up to a nominal strain of 2.5%, and the corresponding macroscopic strain evolution was recorded. A progressive reduction in measured strain is observed for the same nominal substrate deformation after each cycle, along with incomplete relaxation upon flattening, indicating irreversible damage accumulation. Figure S8b shows the recovery of ε_x upon unloading under compressive bending for membranes of different thicknesses, illustrating the dependence on whether the critical strain was reached. When unloaded before reaching the critical strain (top right inset, 20 nm membrane), ε_x returns to zero with negligible hysteresis, indicating elastic and reversible deformation. In contrast, when the critical strain is exceeded before unloading, the strain evolution exhibits a clear hysteresis loop and ε_x

after flattening becomes slightly tensile (bottom left inset), independent of membrane thickness.

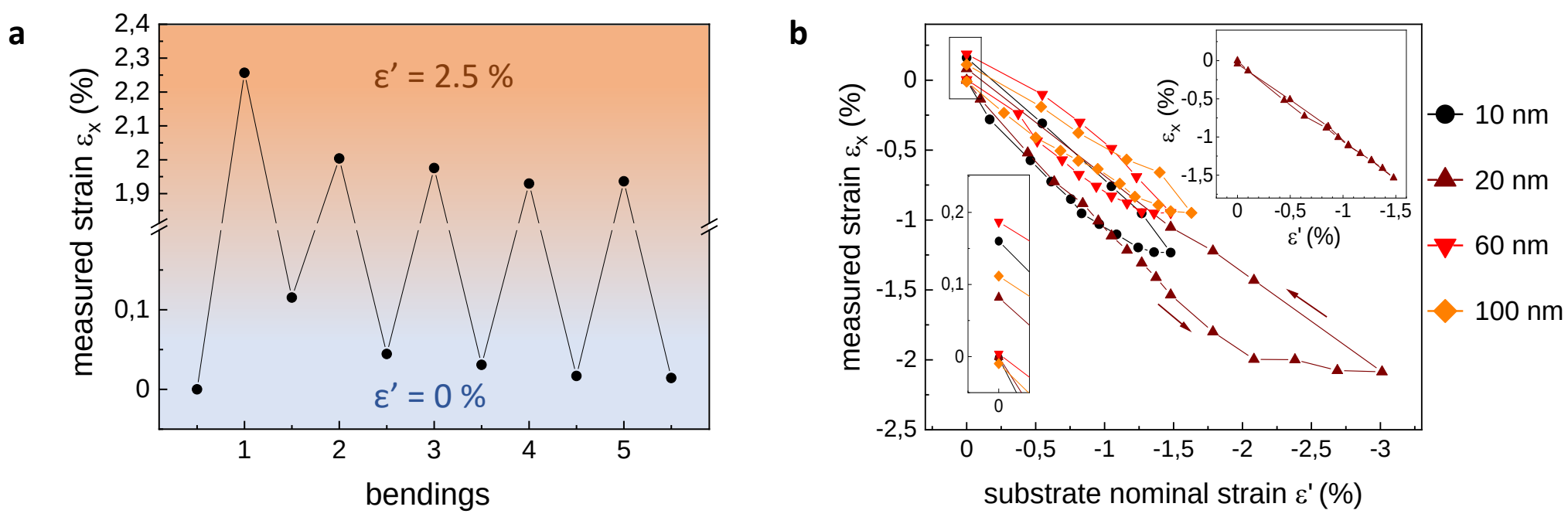

Figure S8: Membrane recovery after unloading. (a) Macroscopic strain ε_x evolution of a 10 nm LSMO membrane subjected to five consecutive cycles of tensile bending at a nominal applied strain $\varepsilon' = 2.5\%$. (b) Macroscopic strain ε_x during compressive bending and subsequent unloading for 10, 20, 60, and 100 nm LSMO membranes that were loaded beyond the critical strain. Top right inset: 20 nm LSMO membrane unloaded before reaching the critical strain, showing full elastic recovery. Bottom left inset: residual tensile ε_x values measured at the flat position before loading (pristine) and after unloading, for all thicknesses.

Substrate effect. Efficient strain transfer critically depends on the choice of substrate: proper adhesion and a smooth surface are essential to ensure that the membrane remains clamped during stress application without fracturing.[4] In this work, we systematically used PI sheets[2] as flexible substrate due to its smooth surface (Rms $\approx$ 2.5 nm), good adhesion, and large Young's Modulus ($\sim$2.5 GPa), making it a suitable candidate for efficient strain transfer. Figure S9 illustrates two examples of how the substrate influences macroscopic strain transfer in LSMO membranes subjected to tensile bending. In Fig. S9a, a bare PI sheet is compared to a PI sheet with adhesive (Kapton tape). While stronger adhesion using tape might be expected to improve strain transfer, the rough adhesive layer can damage the thin membrane upon transfer and its smaller Young's Modulus could lower strain efficiency. Indeed, in this case, no measurable strain transfer was achieved on Kapton tape. A second comparison (Fig. S9b) between PI and PEEK sheets shows that strain transfer efficiency can vary substantially with the substrate material. Similar observations were reported in a study of LSMO membranes transferred onto metal-coated polymer substrates (TiN, Au, Pt), where the bonding between the oxide and the underlying metal was shown to dominate the strain transfer efficiency.[5]

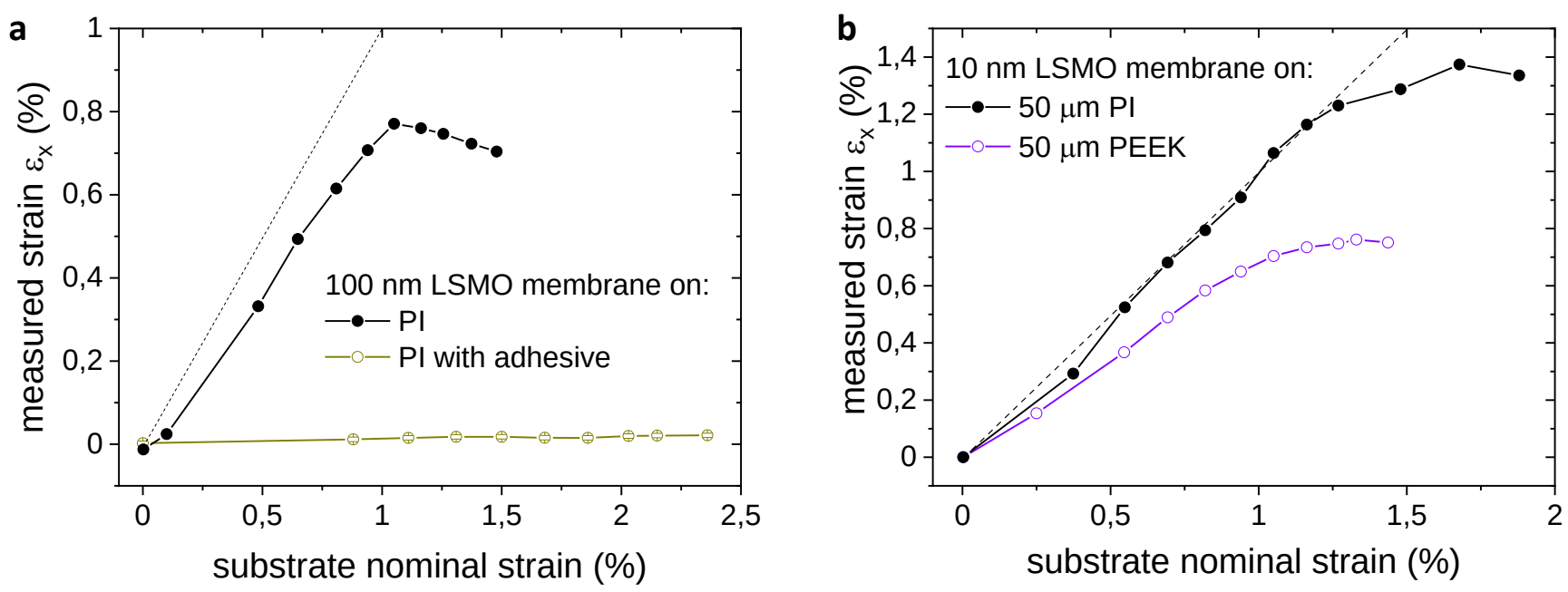

Figure S9: Macroscopic strain ε_x evolution of LSMO membranes under tensile bending on different substrates: (a) 100 nm LSMO membranes on bare PI versus PI with adhesive (Kapton tape), and (b) 10 nm LSMO membranes on bare PI versus PEEK sheets (both 50 μm thick).

Reproducibility and processing effects. Figure S10a shows the measured in-plane macroscopic strain (ε_x and ε_y) for three 10 nm LSMO membranes subjected to tensile bending. Samples A and B originate from the same parent substrate and so were prepared under identical conditions, using a solution-processed $SrCa_2Al_2O_6$ sacrificial layer to release the PLD-grown LSMO,[3] followed by transfer onto the PI substrate. In contrast, Sample C was released from a PLD-grown $Sr_3Al_2O_6$ sacrificial layer.[5] The comparison highlights the significant influence of processing conditions on strain transfer: Sample C exhibits a markedly higher strain transfer than A and B, likely due to its lower defect density, as PLD sacrificial layers generally yield higher crystalline quality than solution-processed ones.[6] Nevertheless, even samples prepared under identical conditions (A and B) present slightly different critical strain of collapse. To assess the reproducibility of critical strain achieved with different mechanical loading modes, Figure S10b shows the critical strain values extracted for two membranes prepared under identical conditions under each stress mode: stretching (20 nm LSMO) and tensile and compressive bending (10 nm LSMO). In all cases, the two nominally identical samples yield different critical strain values, with discrepancies ranging from less than 0.1% under compressive bending to 0.2% under tensile bending. This sample-to-sample variability likely reflects differences in defect density, membrane size and shape, all of which influence the onset of cracking, underscoring the importance of in situ strain measurements, as ex-situ characterizations alone cannot reliably capture the variability between nominally identical membranes.

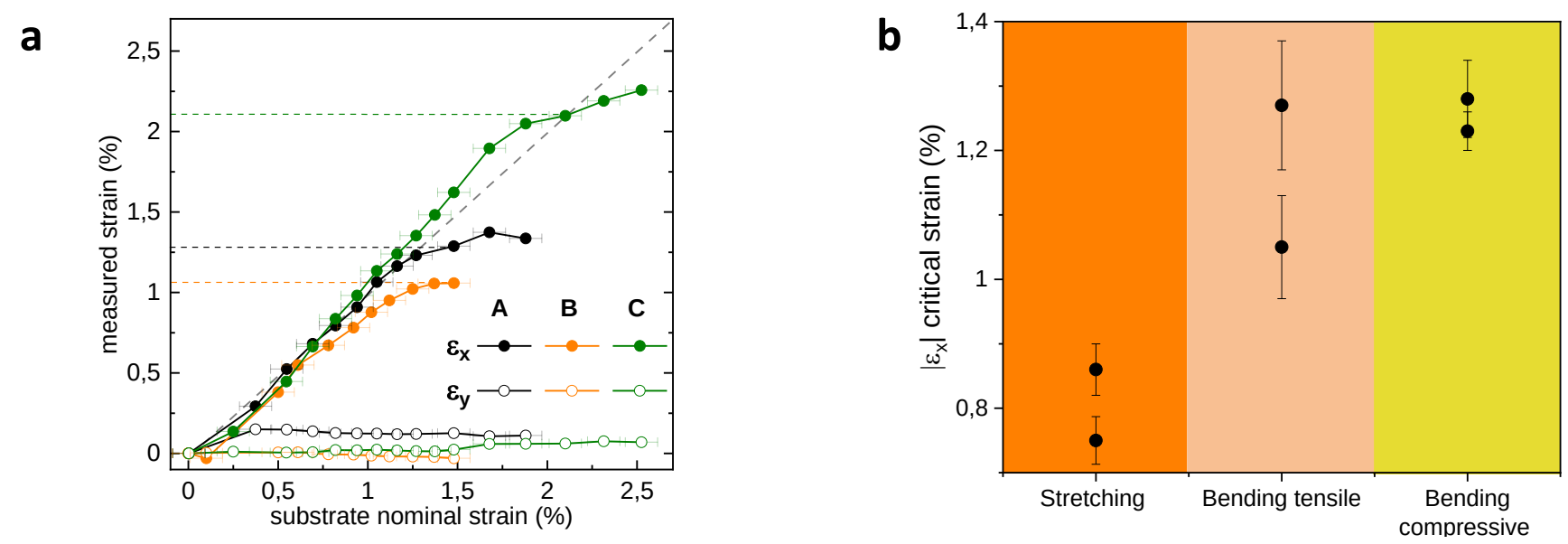

Figure S10: Macroscopic strain evolution of 10 nm LSMO membranes under tensile bending. Samples A and B were prepared under identical conditions (solution-processed $SrCa_2Al_2O_6$ sacrificial layer), while sample C was fabricated using a PLD-grown $Sr_3Al_2O_6$ sacrificial layer. (b) Critical strain values extracted for pairs of nominally identical membranes measured under stretching (20 nm LSMO) and tensile and compressive bending (10 nm LSMO). Error bars represent the uncertainty in the critical strain determination.

Spatially resolved strain mapping

60 nm LSMO membrane under stretching. Figure S11 shows the spatially resolved analysis of a 60 nm LSMO membrane under stretching. The spatially resolved strain maps (Fig. S11b,c) present a mean value in agreement with the macroscopic trends (Fig. S11a), but with increased strain heterogeneity as the applied stress increases. This effect is also clearly captured in the strain population distributions (Fig. S11f,g). The microstrain, extracted from the FWHM of Q(200) (Fig. S11d), follows the same trend as in thinner films, with an initial decrease and then higher microstrain when the applied stress is close to the membrane's collapse.

The spatially resolved orientation maps in χ reveal that, starting from a rather uniform orientation, a distinct reorientation pattern develops progressively as strain increases. Here, the mapped region corresponds to the bottom part of a rectangular-shaped membrane. At $\varepsilon' = 3.0\%$, the orientation map (Fig. S11e-bottom) reveals a clear redistribution of χ, with a symmetry emerging from the sample corners and the center of the membrane. Notably, comparison with the corresponding strain maps (Fig. S11b,c bottom) shows that regions along the reorientation bands and at the membrane edges exhibit systematically lower strain transfer than areas with homogeneous orientation (small strain transfer corresponds to bluish contrast in ε_x and reddish contrast in ε_y). This behavior can be understood by considering the membrane as a thin elastic sheet: at reorientation zones, part of the applied deformation is accommodated through lattice rotation or partial relaxation rather

than pure tensile strain. As a result, these regions act as mechanical buffers that redistribute strain, leading to the observed correlation between orientation patterns and local strain variations.

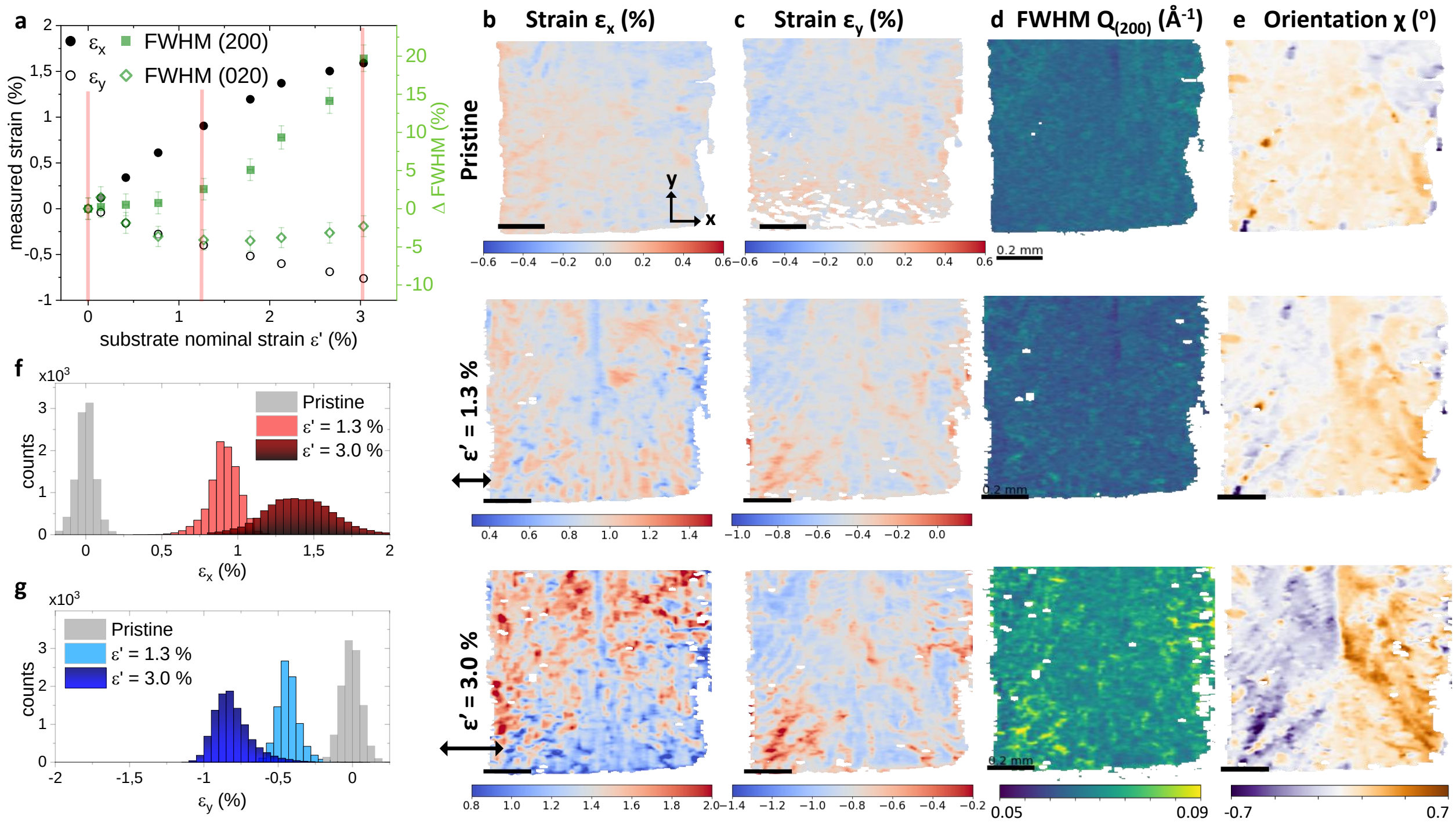

Figure S11: Spatially resolved analysis of a 60 nm LSMO membrane under stretching. (a) Macroscopic strain evolution and FWHM broadening of the (200) and (020) in-plane reflections as a function of applied nominal strain. The nominal strain values selected for mapping are indicated by red lines. Spatial maps of (b) ε_x, (c) ε_y, (d) (200) FWHM in Q (Å^{-1}), and (e) orientation χ are shown for the pristine state (top), $\varepsilon' = 1.3\%$ (middle), and $\varepsilon' = 3.0\%$ (bottom). The strain maps use a mean-centered color scale with a fixed range of 1.2 %. The scale bars are 0.2 mm. The corresponding strain distributions are plotted in (f) ε_x and (g) ε_y.

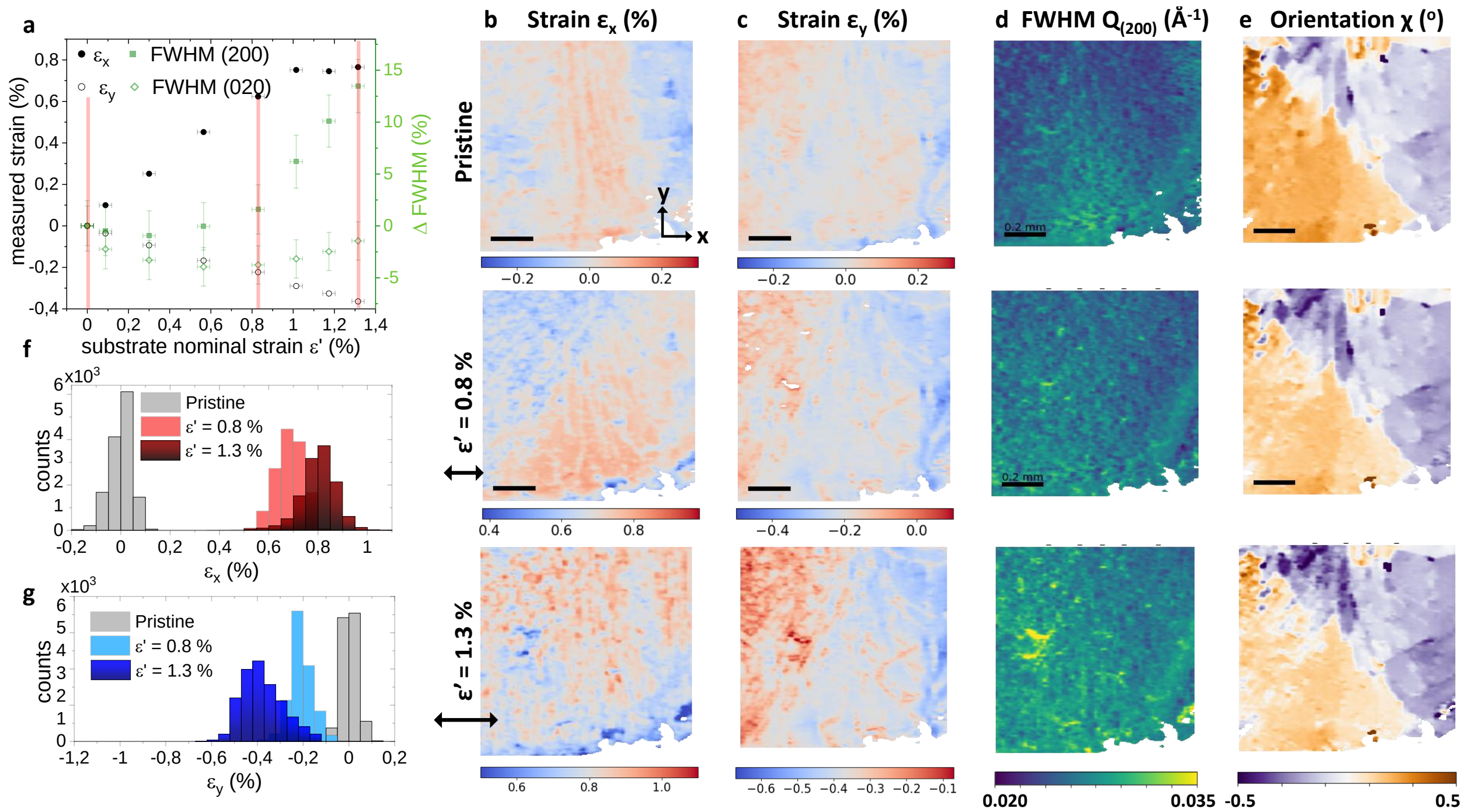

Figure S12: Spatially resolved analysis of a 100 nm LSMO membrane under stretching. (a) Macroscopic strain evolution and FWHM broadening of the (200) and (020) in-plane reflections as a function of applied nominal strain. The nominal strain values selected for spatial mapping are indicated by red lines. Maps are shown for (b) strain in a-LP (ε_x), (c) strain in b-LP (ε_y), (d) (200) FWHM in Q (Å^{-1}), and (e) Orientation in χ, each presented for the pristine state (top), $\varepsilon' = 0.8$ % (middle), and $\varepsilon' = 1.3$ % (bottom). The strain maps use a mean-centered color scale with a fixed range of 0.6 %. The scale bars are 0.2 mm. The corresponding strain distributions extracted from the mappings are plotted for (f) ε_x and (g) ε_y.

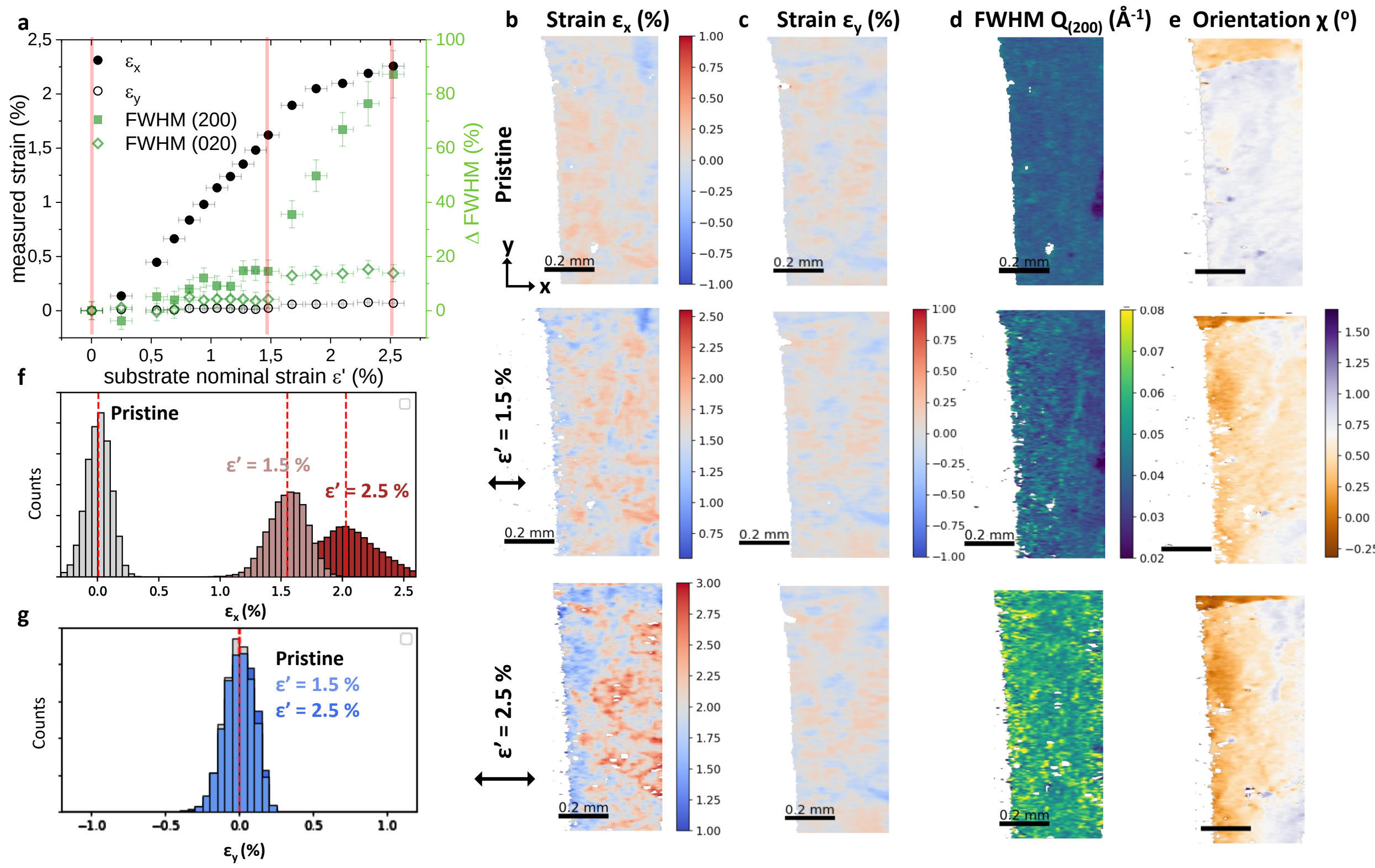

Figure S13: Spatially resolved analysis of a 10 nm LSMO membrane under tensile bending. (a) Macroscopic strain evolution and FWHM broadening of the (200) and (020) in-plane reflections as a function of applied nominal strain. The nominal strain values selected for spatial mapping are indicated by red lines. Maps are shown for (b) strain in a-LP (ε_x), (c) strain in b-LP (ε_y), (d) (200) FWHM in Q (Å^{-1}), and (e) Orientation in χ, each presented for the pristine state (top), $\varepsilon' = 1.5$ % (middle), and $\varepsilon' = 2.5\%$ (bottom). The strain maps use a mean-centered color scale with a fixed range of 2 %. The scale bars are 0.2 mm. The corresponding strain distributions extracted from the mappings are plotted for (f) ε_x and (g) ε_y.

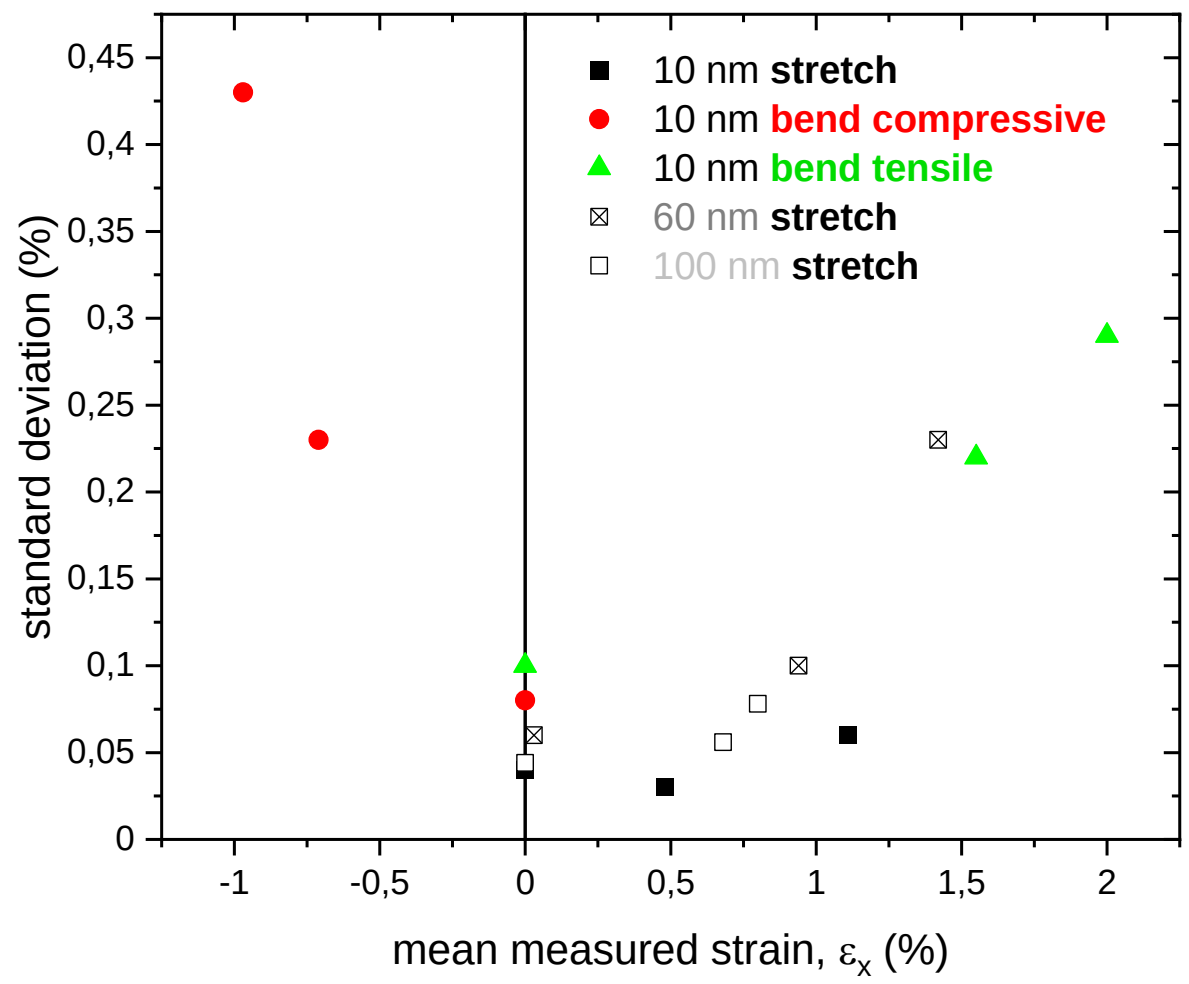

Figure S14: Strain statistics extracted from the spatially resolved strain mapping of LSMO membranes of 10, 60 and 100 nm under different stress modes: standard deviation calculated from the strain distribution population as a function of the mean measured strain ε_x.

Estimation of the effect of strain heterogeneity on T_C. To estimate how the experimentally measured strain heterogeneity could affect the Curie temperature (T_C) of LSMO, we employed a simplified approach previously used for biaxially strained films. Originally proposed by Millis *et al.*[7] and then adapted by others,[8–11] the Curie temperature depends on both the bulk and biaxial strain components according to

$$T_C = T_{C0}\left(1 + \alpha\,\varepsilon_B - \frac{\Delta}{2}(\varepsilon^*)^2\right), \tag{2}$$

For simply biaxial strained films ($\varepsilon_x = \varepsilon_y$), the bulk compression is $\varepsilon_B = -(2\varepsilon_x + \varepsilon_z)$ and the biaxial distortion is $\varepsilon^* = \frac{1}{2}(\varepsilon_z - \varepsilon_x)$. In our case, the system experiences a non-symmetric biaxial strain, with ε_x, ε_y, and ε_z differing due to uniaxial mechanical loading. To account for this, the equation was adapted from the initial Millis's definition $\varepsilon_B = -(\varepsilon_x + \varepsilon_y + \varepsilon_z)$ and $\varepsilon^{*2} = (\frac{1}{2}(\varepsilon_x - \varepsilon_y))^2 + (\frac{1}{4}(2\varepsilon_z - \varepsilon_x - \varepsilon_y))^2$ yielding

$$T_C = T_{C0}\left[1 - \alpha\,(\varepsilon_x + \varepsilon_y + \varepsilon_z) - \Delta\left(\frac{(\varepsilon_x - \varepsilon_y)^2}{3} + \frac{(2\varepsilon_z - \varepsilon_x - \varepsilon_y)^2}{12}\right)\right], \tag{3}$$

The parameters α and Δ (defined as $\alpha = \frac{1}{T_C} dT_C/d\varepsilon_B$ and $\Delta = \frac{1}{T_C} d^2T_C/d(\varepsilon^*)^2$) correspond to ε_B and ε^* and can, in principle, be determined experimentally. We adopted the values reported by Adamo et al., who calculated $T_{C0} = 345$ K, $\alpha = 1.55$, and $\Delta = 1460$ for 22 nm thick LSMO films on various substrates, with a biaxial strain range of -2.3% to +3.2%.[11] However, literature reports significant variability in these parameters depending on LSMO stoichiometry and film thickness, among other factors.[7–11] Therefore, this approach should be regarded as a qualitative, illustrative estimate of the magnitude of T_C variations expected from the observed strain heterogeneity, rather than a quantitative prediction, and direct magnetic measurements would be required for more accurate determination. The in-plane strains ε_x and ε_y were taken from HRXRD measurements, while the out-of-plane strain ε_z was estimated using Poisson's law (Figure S4), assuming isotropic elasticity with a Poisson ratio of $\nu = 0.37$. We note that ε_z was not directly accessible in our experimental geometry, and the assumption of isotropic elasticity may not fully capture the anisotropic elastic response of LSMO. This introduces additional uncertainty into the T_C estimate. The results are presented in Figure 5.

Structural characterization of LSMO films and membranes by XRD

Prior to the *in situ* synchrotron XRD study, macroscopic structural characterization was performed by laboratory-scale XRD on both the as-grown heterostructures and the released membranes. Wide-range θ–2θ XRD patterns of the as-grown heterostructures (Figure S15a) confirm the phase purity of the LSMO films and the absence of secondary crystalline phases across the full angular range ($2\theta = 20°$–$80°$). Reflections are consistent with a perovskite structure and index to the pseudocubic unit cell of $La_{0.7}Sr_{0.3}MnO_3$, alongside the sacrificial layer and the STO substrate peaks. The two synthesis routes explored in this work — LSMO deposited by PLD on a PLD-grown $Sr_3Al_2O_6$ (SAO) sacrificial layer (LSMO/PLD-SAO//STO), and LSMO deposited by PLD on a CSD-grown $SrCa_2Al_2O_6$ (SCAO) sacrificial layer (LSMO/CSD-SCAO//STO) — both yield phase-pure LSMO films with no detectable secondary phases.

Figure S15b shows a zoom around the STO(002) reflection, where the LSMO(002) peak and the sacrificial layer SAO(008) or SCAO(008) are resolved. For fully relaxed LSMO (pseudocubic a = 3.876 Å), the expected position of the (002) reflection is indicated by a dashed line. In the as-grown heterostructures, the LSMO(002) peak appears at higher 2θ values than the relaxed reference, when grown on PLD-SAO, indicating tensile in-plane strain imposed by the sacrificial layer, which forces the LSMO film to contract out-of-plane (Poisson effect), while it appears at lower 2θ values when grown on the CSD-SCAO sacrificial layer, indicating compressive in-plane and tensile out-of-plane strain. The degree of strain varies with LSMO film thickness, being less strained when the LSMO is thicker, probably associated with a more defective and partially relaxed structure.

On the other hand, after sacrificial layer etching and transfer onto PI substrates (Figure S15c), the LSMO(002) reflection shifts toward the fully relaxed LSMO position (dashed line) for all samples, regardless of the original heterostructure route or film thickness. This indicates that epitaxial strain is largely released upon membrane lift-off, and that the membranes recover lattice parameters close to those of stoichiometric $La_{0.7}Sr_{0.3}MnO_3$ prior to any applied mechanical deformation. No systematic difference in the degree of strain relaxation is observed between membranes originating from SAO or SCAO sacrificial layers, suggesting that the choice of sacrificial layer does not introduce a macroscopically measurable residual strain state in the released membrane.

We note that the diffracted intensities shown in Figures S15 are qualitative and should not be

compared directly across panels. Data were acquired under different instrumental conditions and on samples with fundamentally different geometries (thin film on a rigid crystalline substrate vs. small pieces of membrane on an amorphous polymer support), which strongly affect peak intensities and backgrounds. LSMO/PLD-SAO//STO heterostructures and the corresponding membranes were measured using a Rigaku SmartLab diffractometer with high-resolution parallel beam optics and monochromatic Cu-Kα_1 radiation, while LSMO/CSD-SCAO//STO heterostructures and membranes were measured on a Bruker-AXS D8 Discover (A25) also with monochromatic Cu-Kα_1 radiation. For the thinnest membranes studied (10 nm LSMO), the LSMO(002) reflection falls below the detection limit under the measurement conditions used, and is therefore not resolved in the patterns shown.

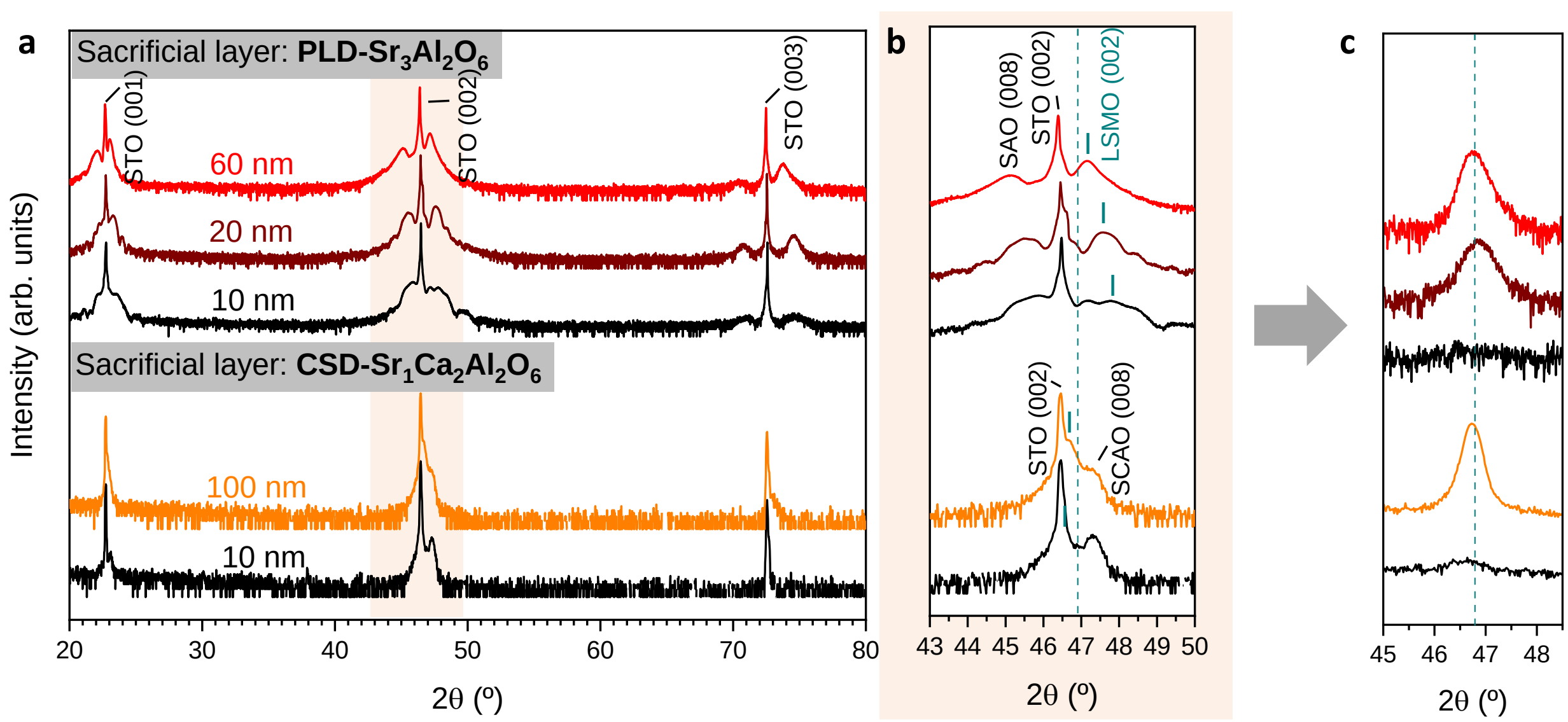

Figure S15: XRD characterization of LSMO thin films and membranes. (a) Wide-range θ–2θ XRD patterns of the as-grown heterostructures prior to sacrificial layer etching, for both synthesis routes: LSMO/PLD-SAO//STO and LSMO/CSD-SCAO//STO. Substrate (STO) reflections are indexed. (b) Zoom of the region around the STO(002) reflection, showing the LSMO(002) peak position as a function of film thickness and sacrificial layer type for the as-grown heterostructures. The vertical dashed line indicates the expected 2θ position for fully relaxed LSMO (pseudocubic a = 3.876 Å). (c) Corresponding LSMO membranes after sacrificial layer etching and transfer onto PI substrates. Note that diffracted intensities are qualitative and not directly comparable across panels, as data were collected under different instrumental conditions, in different laboratories and on samples with different geometries (film-on-substrate vs. membrane-on-PI).

References

(1) Chiabrera, F. M.; Yun, S.; Li, Y.; Dahm, R. T.; Zhang, H.; Kirchert, C. K. R.; Christensen, D. V.; Trier, F.; Jespersen, T. S.; Pryds, N. Freestanding Perovskite Oxide Films: Synthesis, Challenges, and Properties. *Annalen der Physik* **2022**, 2200084.

(2) DuPont de Nemours, Inc. DuPont® Kapton® HN Polyimide Film – Product Specifications (Datasheet). 2024; `https://www.dupont.com/content/dam/electronics/amer/us/en/electronics/public/documents/en/EI-10206-Kapton-HN-Data-Sheet.pdf`, Datasheet no. EI-10206, revision 7/24.

(3) Salles, P.; Guzman, R.; Barrera, A.; Ramis, M.; Caicedo, J. M.; Palau, A.; Zhou, W.; Coll, M. On the Role of the $Sr_{3-x}Ca_xAl_2O_6$ Sacrificial Layer Composition in Epitaxial $La_{0.7}Sr_{0.3}MnO_3$ Membranes. *Advanced Functional Materials* **2023**, *33*, 2304059.

(4) Moon, J.-Y.; Bae, S.; Ryu, J.; Kim, S.-I.; Han, S.; Kim, J. S.; Choi, J.; Kim, S.; Lee, J.-H.; Choi, S.-G.; Liu, T.-R.; Ahn, S.; Seo, J.; Choi, J.-H.; Kwun, H. J.; Shao, Y.-T.; Kim, H.-D.; Park, J.-H.; Lee, J.-W.; Park, J.-W.; Lee, J.-H.; Ahn, J.-H.; Bae, S.-H. Crack-Free Transfer of Wafer-Scale Freestanding Single-Crystalline Nanomembranes Enabled by Elastically Graded Polymer. *Advanced Materials* **2025**, *early view*, e13080.

(5) Brand, E.; Salles, P.; Palliotto, A.; Wang, H.; Chen, W.; Dollekamp, E.; Ramis, M.; Li, Q.; Drnec, J.; García-Lastra, J. M.; Coll, M.; Pryds, N.; Park, D.-S. Strain Engineering of Complex Oxide Membranes on Flexible Metallic Support. *Advanced Physics Research* **2025**, *4*, e00075.

(6) Salles, P.; Caño, I.; Guzman, R.; Dore, C.; Mihi, A.; Zhou, W.; Coll, M. Facile Chemical Route to Prepare Water Soluble Epitaxial $Sr_3Al_2O_6$ Sacrificial Layers for Free-Standing Oxides. *Advanced Materials Interfaces* **2021**, *8*, 2001643.

(7) Millis, A. J.; Darling, T.; Migliori, A. Quantifying strain dependence in "colossal" magnetoresistance manganites. *Journal of Applied Physics* **1998**, *83*, 1588–1591.

(8) Tsui, F.; Smoak, M. C.; Nath, T. K.; Eom, C. B. Strain-dependent magnetic phase diagram of epitaxial $La_{0.67}Sr_{0.33}MnO_3$ thin films. *Applied Physics Letters* **2000**, *76*, 2421–2423.

(9) Ranno, L.; Llobet, A.; Tiron, R.; Favre-Nicolin, E. Strain-induced magnetic anisotropy in epitaxial manganite films. *Applied Surface Science* **2002**, *188*, 170–175.

(10) Thiele, C. Influence of strain on the magnetization and magnetoelectric effect in $La_{0.7}A_{0.3}MnO_3$/PMN-PT(001) (A=Sr,Ca). *Physical Review B* **2007**, *75*, 054408.

(11) Adamo, C.; Ke, X.; Wang, H. Q.; Xin, H. L.; Heeg, T.; Hawley, M. E.; Zander, W.; Schubert, J.; Schiffer, P.; Muller, D. A.; Maritato, L.; Schlom, D. G. Effect of biaxial strain on the electrical and magnetic properties of (001) $La_{0.7}Sr_{0.3}MnO_3$ thin films. *Applied Physics Letters* **2009**, *95*, 112504.
